# Potential therapeutic strategies for photoreceptor degeneration: the path to restore vision

**DOI:** 10.1186/s12967-022-03738-4

**Published:** 2022-12-07

**Authors:** Fereshteh Karamali, Sanaz Behtaj, Shahnaz Babaei-Abraki, Hanieh Hadady, Atefeh Atefi, Soraya Savoj, Sareh Soroushzadeh, Samaneh Najafian, Mohammad Hossein Nasr Esfahani, Henry Klassen

**Affiliations:** 1 Department of Animal Biotechnology, Cell Science Research Center, Royan Institute for Biotechnology, ACECR, Isfahan, Iran; 2 Clem Jones Centre for Neurobiology and Stem Cell Research, Griffith University, Queensland, Australia; 3 Menzies Health Institute Queensland, Griffith University, Southport, QLD 4222 Australia; 4 Gavin Herbert Eye Institute, Irvine, CA USA

## Abstract

Photoreceptors (PRs), as the most abundant and light-sensing cells of the neuroretina, are responsible for converting light into electrical signals that can be interpreted by the brain. PR degeneration, including morphological and functional impairment of these cells, causes significant diminution of the retina’s ability to detect light, with consequent loss of vision. Recent findings in ocular regenerative medicine have opened promising avenues to apply neuroprotective therapy, gene therapy, cell replacement therapy, and visual prostheses to the challenge of restoring vision. However, successful visual restoration in the clinical setting requires application of these therapeutic approaches at the appropriate stage of the retinal degeneration. In this review, firstly, we discuss the mechanisms of PR degeneration by focusing on the molecular mechanisms underlying cell death. Subsequently, innovations, recent developments, and promising treatments based on the stage of disorder progression are further explored. Then, the challenges to be addressed before implementation of these therapies in clinical practice are considered. Finally, potential solutions to overcome the current limitations of this growing research area are suggested. Overall, the majority of current treatment modalities are still at an early stage of development and require extensive additional studies, both pre-clinical and clinical, before full restoration of visual function in PR degeneration diseases can be realized.

**Graphical Abstract:**

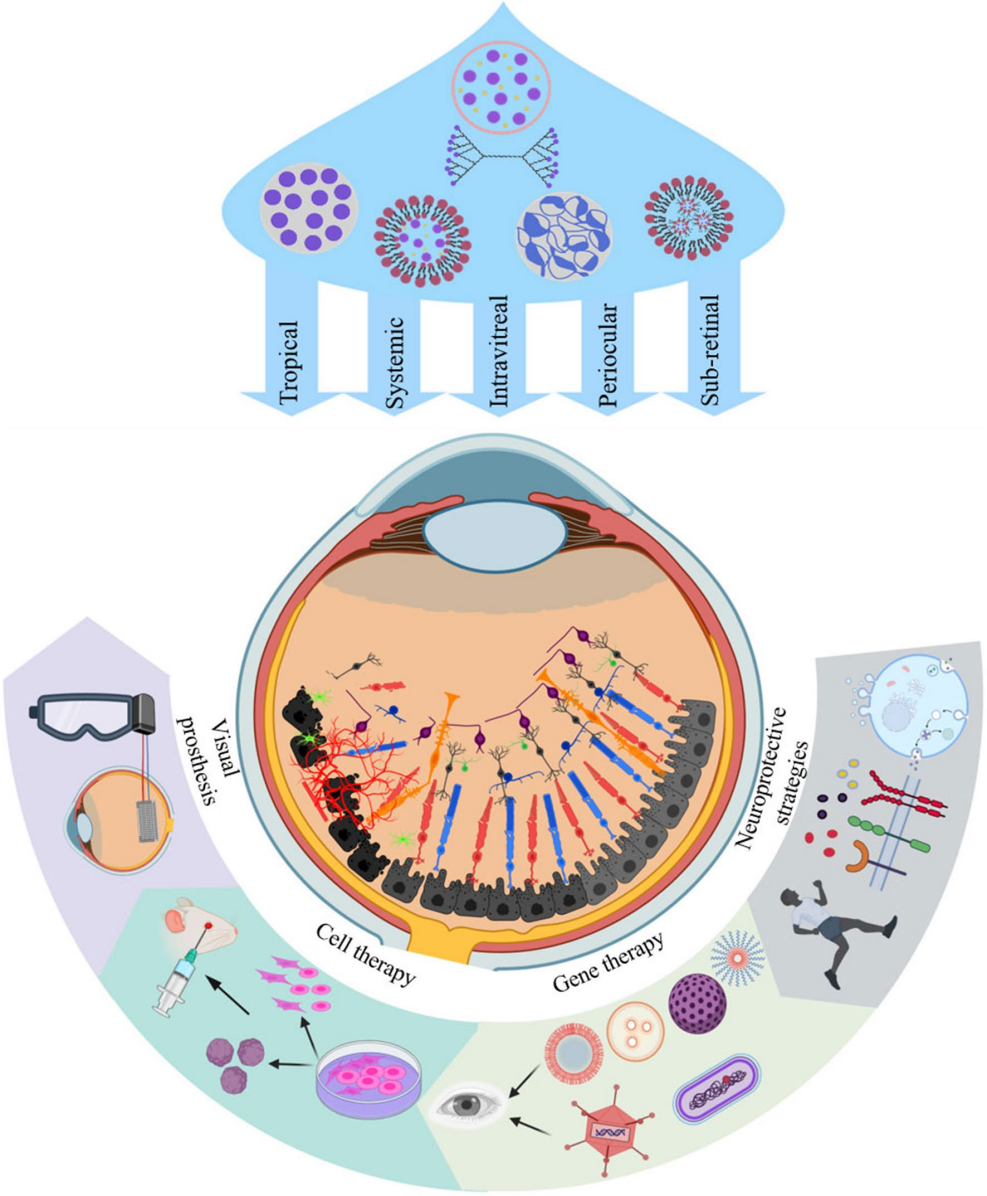

## Photoreceptor degeneration

The neuroretina is a photosensitive membrane located at the posterior of the eye. The light-sensing cells, known as photoreceptors (PRs), are the most numerous and metabolically demanding cells in the retina. Their metabolism is supported by a single-layered retinal pigment epithelium (RPE). PRs are highly polarised retinal neurons, each being organised into several compartments, including the outer segment (OS), the inner segment (IS), the nucleus, and a short axon [1, 2]. These light-sensing cells are classified into rods and cones based on their morphology, as well as their activity profile in bright or dim light. Rod photoreceptors (rods) provide vision under low light conditions, whereas cones are responsible for colour and daylight vision [3]. Loss of PRs and/or their functionalities impairs the ability to detect light as in inherited and acquired PR degenerations [4, 5]. The former reveals itself during childhood while the latter appears later in life possible due to environmental stresses, drug toxicity, and ageing [4].

Age-related macular degeneration (AMD) is the most common form of acquired PR degeneration [3]. In contrast, proliferative vitreoretinopathy (PVR) is another type of common retinal pathology that occurs following retinal detachment and can involve PR degeneration, although that is secondary to the causative mechanism [6]. The inherited forms of PR degeneration are induced by mutations in one, or more, of the many identified or unidentified genes and loci. Until now, more than 300 different genes have been identified, and new genes and loci continue to be discovered [7]. Retinitis pigmentosa (RP), achromatopsia, Leber’s congenital amaurosis (LCA), and Stargardt disease (STGD) are prominent forms of inherited retinal diseases (IRD). Visual deficits can be brought about by an acquired or inherited degenerative process endogenous to the retina, as is typical for PR dystrophies, but it is important to note that RPE-associated processes can also play a role, as in AMD [8]. As loss of vision in inherited and acquired PR degeneration are inflicted through different molecular mechanisms, identifying the specific mechanisms involved could provide new therapeutic approaches for the treatment of retinal degeneration.

## Molecular pathways involved in PR degeneration

There is a growing body of literature implicating the endoplasmic reticulum (ER) and mitochondria in PR cell death [2, 9]. The ER is a critical cellular organelle and plays important functions in many cellular processes such as Ca2 + regulation, as well as protein synthesis, maturation, and folding [10]. Accumulation of misfolded protein in the ER culminates in a process called unfolded protein responses (UPR) [4, 11] and has been reported in several animal models of PR degeneration [12, 13]. In addition to activation of UPR, increased intracellular calcium ions result in cell death through the activation of the cysteine protease calpain, which has been considered an alternative pathway for PR degeneration. Calpain I and II are expressed in the retina and are controlled in vivo by the endogenous tissue inhibitor calpastatin [14, 15]. Activation of calpains requires calpastatin dissociation and translocation to the cytosolic side of the ER [16]. The cell death effector in PRs triggered by calpains is apoptosis-inducing factor (AIF) which is a mitochondrial flavoprotein located in the outer mitochondrial membrane and triggers cell death in a caspase-independent manner. DNA fragmentation is a consequence of proteolytic cleavage and release of AIF from the mitochondria through pores formed by BAX oligomerization [16, 17].

Also, photon absorption by rhodopsin (RHO) leads to a conformational change in RHO protein and allows it to activate transducin and phosphodiesterase-6 (PDE6). PDE6 hydrolyses cGMP to GMP, resulting in diminution of cyclic nucleotide-gated channel (CNGC) permeability, and consequently a reduction of Ca^2+^ influx. Also, it has been demonstrated that high intracellular Ca^2+^ levels resulting from CNGC activation could promote PR degeneration. On the other hand, cGMP elevation results in the activation of cGMP-dependent protein kinase enzymes that trigger the cell death mechanism via a calpain-dependent pathway [18]. Therefore, a large number of different malfunctions throughout RHO can cause protein sequestration in the ER. Thus, modulation of ER stress is also considered as a candidate for treatment of retinal degenerative diseases [19].

## Stages of photoreceptor degeneration

**Fig. 1:**
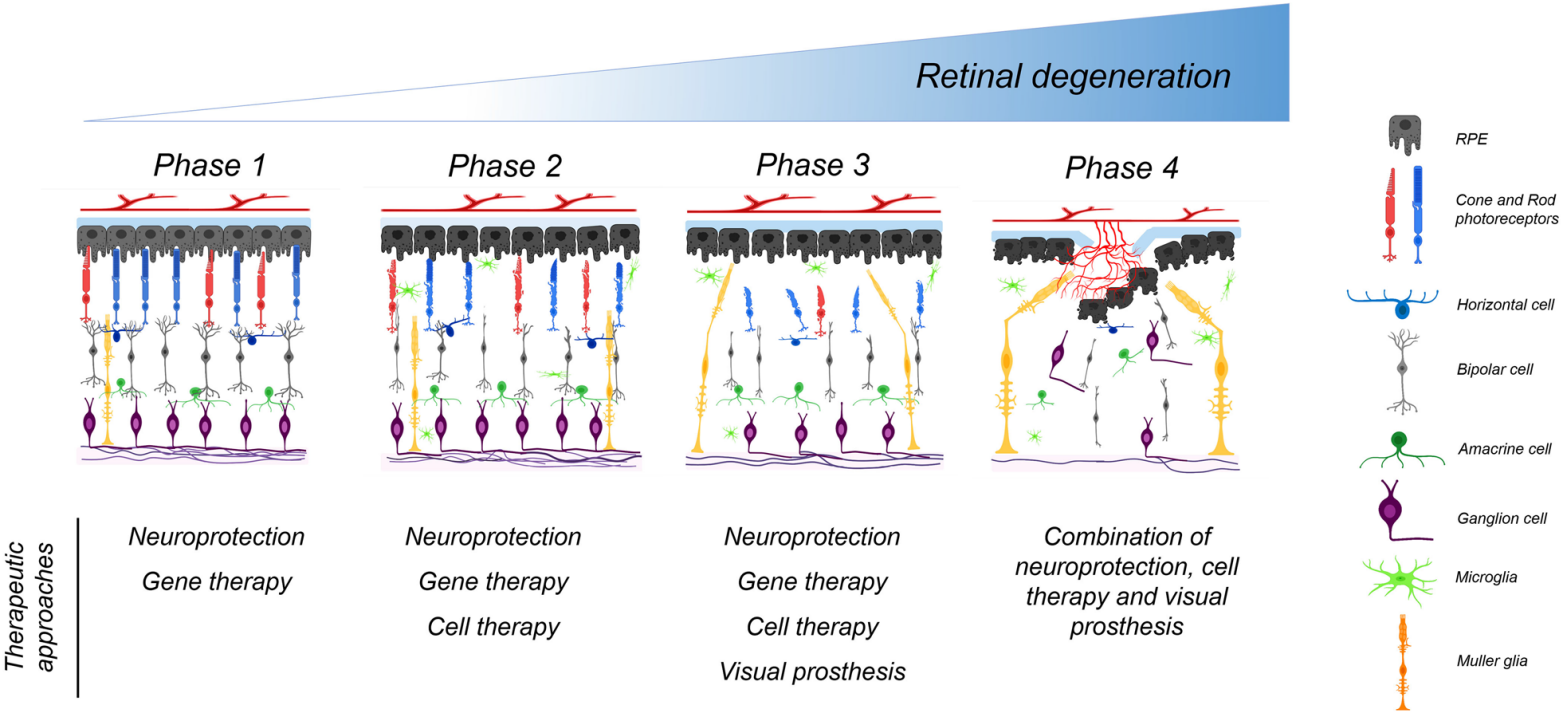
Progression of retinal degeneration. The healthy retina consists of five major classes of neurons: photoreceptors, bipolar cells, retinal ganglion cells, horizontal cells, and amacrine cells, as well as the non-neuronal pigment epithelium. The early, intermediate, advanced, and late-stage of the retinal degenerative process results in changes in the function and morphology of the retina over time. These changes include truncation of the outer segments of PRs, reduction in cell numbers due to cell degeneration and death, appearance of reactive glial cells, hypertrophy of Müller cells, migration of neuronal cells, translocation of amacrine and bipolar cells into other layers, deep synaptic change, cell death progresses, the absence of visual capacity, deterioration of blood-retinal barrier and disruption of RPE and Brunch's membrane. The current therapeutic approaches have also been presented for each degeneration phase

Photoreceptor degeneration is a complicated process with different genes and factors involved, spanning environmental factors to monogenic disorders. Various therapeutic approaches have been adopted in an effort to maintain retinal function or restore vision in pathological conditions [20, 21]. To this end, a detailed understanding of the different phases of retinal degeneration has in turn paved the way for better regenerative approaches (Fig. 1) [22].

In the first stage of retinal degeneration, these conditions can be difficult to recognize, as the PR function and morphology may remain typical. In this stage, whearse the appearance of retina layers may seem to be normal, several changes occur such as the disruption of the RPE in AMD or alteration in glucose concentration in DR. Initial disruption does not necessarily alter the function and morphology of PRs or retinal neurons [23]. Also, there is a phenomenon called glial reactivity (or gliosis), which is a cellular response of Müller glia and astrocytes to a different form of retinal damages [24]. Gliosis starts in the early stages of photoreceptor degeneration and may lead to glial seals and scars in the advanced retinal deterioration [25]. After gliosis, glial seals which are formed by Müller cells may complicate the cell therapy and replacement of lost photoreceptors by affecting the migration of transplanted cells thereby limiting the formation of new synaptic connections [26–28]. It is important to note that the remaining cells are in a good structure and shape to enhance the chance of success in a cell-based therapy via a selective cell type like photoreceptors. It has been demonstrated that the following in progression in reactive gliosis, glial processes may extend into the vitreous side and subsequently form fibrotic scar which may result in retinal detachments and thereby hindering tissue regeneration [29–31]. So, other options such as retinal implants should be selected. Although, glial scar formation can delay the chance of success through the formation of a physical barrier between implants and host neuroretina [32–34]. This issue makes therapies to choose other treatments like preserving the remaining cells with neurotrophic factors to restore vision and improve blindness. Overall, not only knowledge about the status of retinal degeneration but also finding the appropriate therapy helped restore vision.

During the second stage, cellular stress activates the apoptotic pathways and leads to PR cell death. At this stage, delocalization of RHO in the rods and the transduction proteins in cones are considered as one of the first histopathological signs [35]. Cone PRs also undergo decreases in outer segment length [36]. The synaptic terminals are deconstructed in stressed PRs leading to loss of synaptophysin. The loss of synaptic signals leads to various rewiring events, including bipolar and horizontal cell dendritic contraction, converting of synaptic targets by bipolar cells, and abnormal extension of horizontal cell processes into the inner plexiform layer (IPL) [37]. Also, ocular degeneration causes a cascade of actions during the early stages of PR dysfunction that terminate in molecular changes. In particular, Ca^2+^ overload has been recently referred to as a damaging process in the early stages of PR degeneration. It is worth noting that high intracellular Ca^2+^ is considered a common mechanism of the degeneration process in general, as reported in mutant animal models [38]. Patients at the early stages of the disease still have PRs: and seek a cure to interrupt the degeneration process in hope of preserving the remaining vision.

Neuroprotection and gene therapy methods are mostly based on neuroprotection with the aim of preventing neuronal degeneration and slowing the progression of the disease by interfering with inflammation, oxidative stress, apoptosis, and delivering drugs to specific survival pathways [39].

During the third phase, although the remaining PRs still preserve a degree of function, they are also engaged in the process of degeneration, and the other cells are also at risk of cell death. Concomitant with events, an increase in the gliosis of müller cells is observed and the number of activated microglial cells has been reported to be higher. At the end of this stage, blood vessels respond to the lack of oxygen by progressively producing new vessels. At this stage, the PR layer will gradually disappear. Translocation of residual cell bodies to other retinal layers occurs at this stage. Bipolar cells are not only different physiologically but also anatomically. In this phase, the bipolar cells physically retract their dendrites and thereby severely alter the morphology of the outer INL.

In the fourth phase, there is a lack of visual function owing to the degeneration impacting essentially all retinal PRs. In this stage, amacrine and bipolar cells migrate into the retinal ganglion cell (RGC) layer and induce the formation of microneuromas. Moreover, synapses are formed between bipolar and ganglion cells [40]. This new rewiring of retinal cells results in abnormal visual circuitries. RGCs can migrate into the INL. The hypertrophy and migration of Müller cells generate a complicated lamination of the inner nuclear layer (INL) and outer plexiform layer (OPL). Rods and cones are in the process of being entirely lost at very advanced retinal degeneration stages, with activation of cell stress and apoptosis pathways acting on any residual PRs at this stage [41]. Retinas show dramatic changes in morphology [42], where rewiring of the retina is extensive with neurite extension by all types of neurons in the setting of widespread cell death [43]. When the unavoidable chain of degeneration reaches to the INL, the RGC loss happens and it causes the poor results of therapies. Formation of subretinal vascular complexes is one the latest events occur during wide RP death [44]. Following PR degeneration, the retina shows the high level of hyperoxia which supresses the VEGF secretion [45]. Also, the breakdown of blood- retina barrier [44] and disorganization of RPE layer via new vasculture complex, leads to irreversible retinal diorders which affect the final results of therapies [46].

## Therapeutic approach: points to consider

The eye position, within the orbital bones and associated tissue, is in a protective location, which also acts as an anatomical barrier and separates it from the rest of the body to a degree. The eyeball itself consists of an anterior segment, extending from the cornea to the lens, and a posterior segment extending from the lens to the retina, which is composed of the vitreous humour, sclera, choroid, retina, and optic nerve [47]. The various ocular barriers ranging from static (membranous) to dynamic (vascular) barriers, which are caused by the complex anatomy and physiology of the eye, limit the efficacy of approaches that require delivery to the posterior segment of the eye, including the retinal PRs. The static natural barriers consist of corneal epithelium, sclera, choroid, Brunch’s membrane, RPE, and conjunctiva. The dynamic barriers (i.e., blood-aqueous barrier and blood-retinal barrier) contain choroidal and conjunctival blood flow, lacrimation, and lymphatic drainage and efflux [48, 49]. All these ocular barriers limit therapeutic approaches, especially for the delivery of any substance or elements to the retina [50]. Even though there has been much research into intraocular delivery, such as vehicles and administration techniques, effective delivery to specific target sites within the posterior segment remains particularly challenging [51]. The majority of this review focuses on two main areas: administration routes and carrier systems.

### Administration routes

**Fig. 2:**
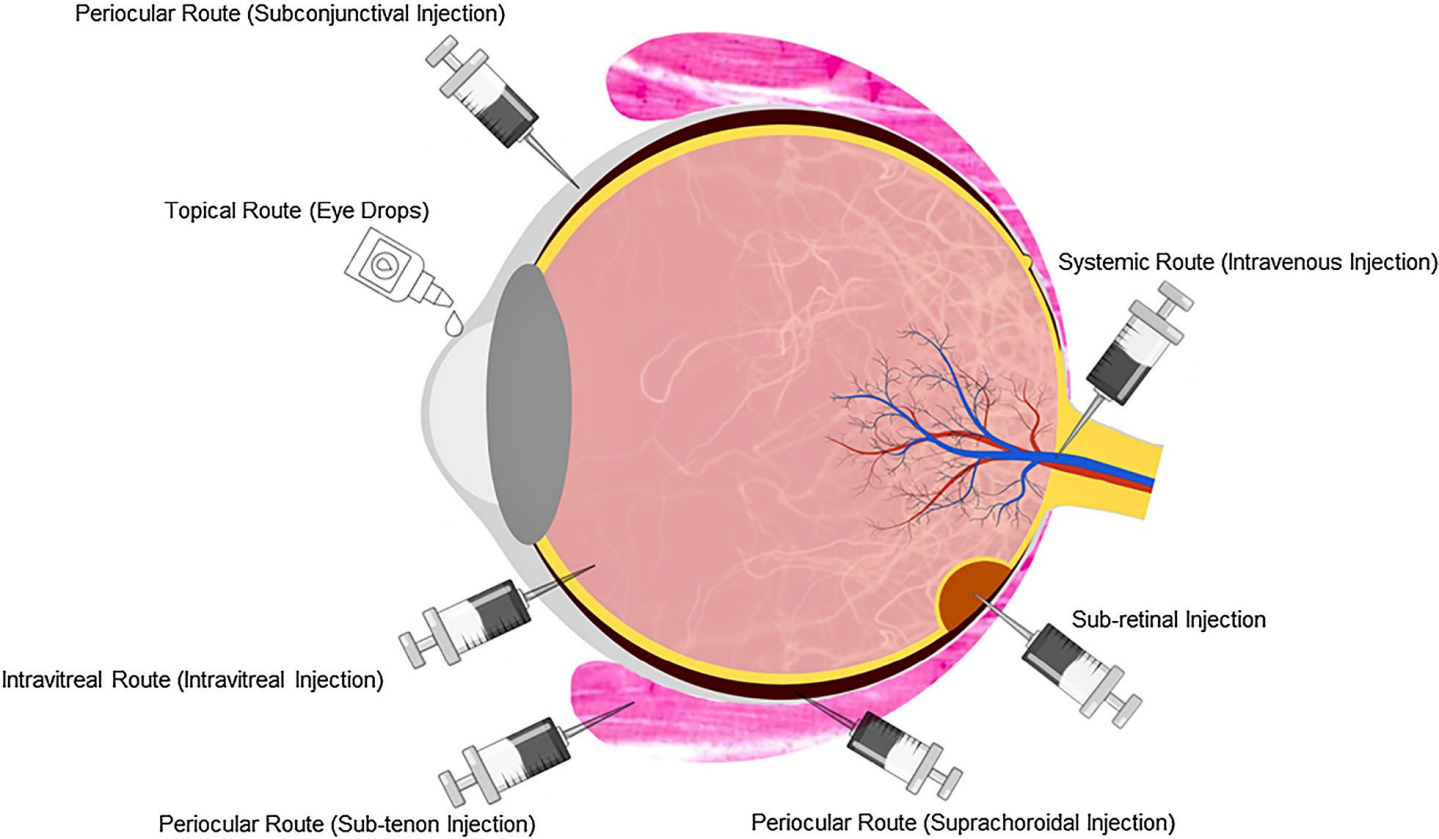
Schematic presentation of various routes for drug delivery to retina, including topical, intravitreal, systemic, and periocular and sub-retinal routes

Administration routes are commonly defined as the way that therapeutic elements, including pharmacologically active agents, trophic factors, gene therapy agents, or donor cells can be delivered to the target area. The identification of appropriate routes in these deliveries has been a focus of recent investigations. In this review, a summary of administration routes to the retina, especially the posterior segment is provided (Fig. 2).

#### Topical route

Topical ocular delivery, such as eye drops, suspensions, and ointments, is a viable and non-invasive route for administering therapeutics to the eye for the anterior segment. However, the effectiveness of this route to treat posterior segment diseases is limited by the low penetration of therapeutic molecules; thus, less than 5% of the drug reaches the target area [52–54]. Delivery of the pharmacological elements to the posterior segment via topical dosing has been broadly investigated in rodents [48]. However, this treatment, when applied to human subjects, yielded limited success owing to their corneal thickness, aqueous humour volume, flow rate, vitreal volume, and circumferential or linear distance from the ocular surface to the back of the eye [48]. For effective delivery of the topical drugs, three main factors need to be satisfied: sufficient aqueous solubility, lipophilicity, and residence time. Therefore, the clinical success of the delivery to the posterior segment via this route remains elusive and requires further extensive preclinical assessment.

#### Systemic route

In a systemic route, therapeutic elements are administered via intravenous injection. Local delivery to the PRs may potentially be possible by systematic administration of the prodrugs using novel delivery systems, including liposomes or nanocarriers [18]. However, these delivery routes cannot be considered optimal in the treatment of PR degenerations, since only a low percentage of therapeutic agent permeates through the ocular barrier and reaches the posterior segment [55, 56]. This route imposes challenges at both the molecular (i.e., carrier compound side effects and cost-effectiveness of the process) [57] and intracellular levels (i.e., partial blocking of nanoparticle cellular uptake by binding to extracellular proteins and glycosaminoglycans to the nanocarrier surface) [58, 59].

#### Intravitreal route

Intravitreal (IVT) administration is a route via which a substance is introduced directly into the vitreous, from where it can diffuse throughout the posterior segment. IVT, as the most common and local route of delivery, can overcome many ocular barriers and allow delivery to the PRs. Less frequency of administration, depot action, and sustained pharmacologic effect are the advantages of the IVT. However, ocular pain, infection or haemorrhage, vision impairment on repeated use, and diminished control of drug release are potential challenges associated with this delivery route. Besides, IVT injections such as implant insertion require skilled professional execution for administration, and post-injection/implantation care and removal and replacement are costly [55, 60, 61]. This route is also considered as a candidate for gene and cell therapy (Table 1).

**Table1:** Neuroprotective strategies for photoreceptor regeneration with an emphasis on clinical progress

Treatments	Route of administration	Mechanism of action	State of progress towards clinical therapy
Compounds			
TUCDA	Intraperitoneal [162]; intravitreal injection of TUCD encapsulated microspheres [163]; intravitreal injection	Improving photoreceptor function and Structure [162–168], improving protein folding and trafficking [169], reducing oxidative stress and ER stress [167, 170], suppressing the pro-apoptotic P53 protein [171], anti-inflammatory effects [168, 172]	FDA-approved; no active clinical trial on Retina; well-tolerated by humans with few side effects [173, 174]; the optimal dose for animals (500 mg/kg) seems too high for human; systemic delivery is challenging
UDCA	Intravitreal injection	Breakdown of TUDCA to release taurine [175], stabilizing the mitochondrial membrane [176]	FDA-approved; only one trial (NCT02841306) on retina among 151 studies listed in clinicaltrials.gov working on UDCA
MK-801	Intravitreal injection	NMDA receptor blocker [177, 178]	High efficacy compared to other NMDA blockers; side effects, including illusions, and memory deficits [179]
Brimonidine	Intravitreal implant, topical	Alpha-2 adrenergic receptor agonist (Patent#US2001049369), cone neuroprotection [180], decreasing vitreoretinal vascular endothelial growth factor, inhibiting blood– retinal barrier breakdown [148]	12 trials on retina out of 159 studies working on Brimonidine tartrate in clinicaltrials.gov, prescribed to treat glaucoma and ocular hypertension under trade name ALPHAGAN-P; side effects such as conjunctiva hyperemia, allergic conjunctivitis, and ocular pruritus
Tandospirone (AL-8309B)	Topical ocular	5-HT1A receptor agonist (selective serotonin 1A receptor agonist) [181]	One trial completed NCT00890097 Prescribed to treat Dry macular degeneration
Anti-amyloid β antibody (RN6G, Glatiramer acetate)	Intravenous, subcutaneous	Binds and eliminates amyloid β (IV) [182–184]	Three studies on RN6G and six studies on Glatiramer acetate, listed in clinicaltrials.gov and completed on the retina
Corticosteroids	Intravitreal/subconjunctival, intraperitoneal injection, fasting-mediated stress [185], dietary supplement	Promoting myelination [186–188], improving impaired axonal transport[189], increasing BDNF levels [190], reducing microglial and macrophage activity [191, 192], increasing inflammatory cytokine levels (IL-1β, TNF-α, IL-6, COX-2, and the p65 NF- κB subunit) [193], reducing edema, likely via action on aquaporin four expressions [194], reducing excitotoxicity by acting on the GABAA receptor [195, 196] and the NMDA response [197]	FDA-Approved but failed to pass phase III of clinical trials due to lack of methodological information, long term effects in the retina would have to be evaluated
L-DOPA	Topical [198], dietary supplement	Delaying AMD [199], anti-angiogenic [200], maintaining ERG function and photoreceptor cell counts [201], stimulating G protein coupled receptors (GPCR) like GPR143 in RPE [202, 203]	FDA-approved for Parkinson disease, eight studies on retina listed in clinicaltrials.gov; side-effects on eye must be considered, dosage needs to be optimized
GSK812 small molecule	Sustained induction of GDNF via Intravitreal injection of suspension solution instead of aqueous solution	Upregulation of GDNF mRNA (> 1.8-fold) and protein levels (> 2.8-fold) [204]	GDNF induces neuroprotective effects in retinal cells at concentrations as low as 30 nM
Rasagiline (N-propargyl-1-(R)-aminoindan)	Dietary supplement	Selective monoamine oxidase B inhibitor [205], delaying activation of caspase 3 dependent apoptotic pathways and inducing the anti-apoptotic protein Bcl-X_L_ [206]	Already being used for Parkinson disease, one clinical trial (NCT02068625) on Rasagiline under trade name Azilect of patients with Macula-off Retinal Detachment and terminated due to the recruitment difficulties
Mycophenolate mofetil (MMF)	Intraperitoneal injection	Suppressing the cGMP-dependent cytotoxicity for photoreceptor cell death which occurs independently of the presence of hyperphysiological whole retinal cGMP levels [207]	FDA-approved, already being used off-label as an immunomodulatory agent for ocular inflammation treatment [208]
Neurotrophic factors			
CNTF (NTF-501) [209–212]	Intravitreal injection, encapsulated intravitreal implant	Rescue photoreceptor from degeneration, activation of Jak/STAT, PI3K/Akt, and Ras/MAPK cell survival mechanisms [112], BDNF strengthen synaptic transmission following activation of neuronal firing [213–215]	Five studies on retina listed in clinicaltrials.gov; delivering is challenging due to the instability and inability to cross blood-retina barriers, dosage, timing and target tissue need to be established
BDNF [122, 210]			
PEDF [216, 217]			
GDNF [218–220]			
Fibroblast Growth Factor 21 [217, 221]			
LEDGF [222]			
Antioxidants			
Vitamin A, E and C	Hind leg muscle injection [223], dietary supplement	Body’s natural defense mechanisms against oxidative stress	54 studies listed in clinicaltrials.gov, FDA-approved and easy to take; synergic effect of antioxidants combinations needs to be determined, regeneration efficiency needs to be improved
Lutein	Dietary supplement	Preventing neurodegeneration induced by oxidative stress via affecting pathological pathways of inflammatory cytokines, such as IL-6 and angiotensin II signaling [224]	
Docosahexaenoic acid (essential omega-3 FA family member)		DHA, is concentrated in the nervous system, particularly in photoreceptors and synaptic membranes, neuroprotectin D1, the DHA-derived bioactive lipids may be a mediator that promotes homeostatic modulation of cell integrity during photoreceptor renewal [216, 225, 226], delaying apoptosis and promotes differentiation of photoreceptor [227]	
Lyciumbarbarum Polysaccharides (LBP) [228]		Preventing the generation of reactive oxygen species (ROS), decreasing poly (ADP-ribose) polymerase (PARP14)	
Idebenone		Accelerating the recovery of visual acuity in patients with hereditary optic neuropathy (LHON) [229]	
Safranal [230]		Ameliorated the loss of both rods and cones, synaptic contacts between photoreceptors and bipolar or horizontal cells were preserved	One clinical trial ongoing (NCT01278277)
Rehabilitation methods			
Curcumin [1,7-bis (4-hydroxy-3-methoxyphenyl)-1,6-heptadiene-3, 5 dione]	Intraperitoneal injection	Suppressing N-methyl-N-nitrosourea-induced photoreceptor cell apoptosis in Sprague–Dawley rats through inhibition of DNA oxidative stress [231]	Crossing the blood–brain barrier due to its polyphenolic structure (two phenol rings connected by α, β-unsaturated carbonyl groups) [232]; poor solubility, bioavailability, and lack of stability at physiological conditions, degrading into different compounds such as ferulic acid, vanillin, ferulic aldehyde and feruloyl methane [233], adverse effects have seen on RPE at 10 μM dosage [234]
Resveratrol (3,5,4′-trihydroxystilbene)	Intraperitoneal injection, dietary supplement	Preventing photoreceptor cell death by upregulating FoxO family protein levels (FoxO1a, FoxO3a, FoxO4) and blocking caspase 3, 8 and, 9 activation [235, 236]	Natural compound, one clinical trial (NCT02625376) on age-related Macular Degeneration
Di-apocarotenoid norbixin (BIO201)	Systemic administrations Intraperitoneal injection	Reducing ocular A2E and lipofuscin accumulation [237]	Natural compound, well tolerated by human and animal
Exercise	N/a	BDNF/TrkB signal transduction pathway [238], increasing BDNF [239, 240], blocking TrkB pathway activation using ANA-12, a TrkB inhibitor (K252a) [241, 242]	Five completed studies, voluntary exercise has been more beneficial than forced exercise for animal models[243]; intensity and duration must be optimized for each patient with a specific stage of degeneration; specialized equipment must be utilized for a patient with low vision
Electrical Stimulation [244–248]	Transcorneal	Preserving function and structure, upregulation of neurotrophic factors;	Not FDA-approved Completed clinical trials showing vision improvement (NCT02019927)
Gene therapy			
Block of p75NTR, Lack of p75NTR [249]	Viral transgene	Preventing bFGF reduction, improving structural and functional photoreceptors survival	Clinical studies have been completed on Alzheimer disease
APL-2	Intravitreal injection	A complement C3 inhibitor,	One Clinical trial (NCT03525613) ongoing
Lampalizumab [250]		An antibody against Complement factor D (CFD)	One clinical trial (NCT02247479) terminated
Metformin [251]		Activating adenosine monophosphate-activated protein kinase (AMPK), increasing mitochondrial biogenesis, reducing oxidative stress, reprogramming of metabolism	One Clinical trial (NCT02684578) ongoing
Adeno-associated viral vector serotype 1	Intramuscular injection	Expressing human proinsulin	Requirement for repeated administration can be overcome; gene therapy vectors typically lead to insufficient photoreceptor transduction, may pathologically alter the retina environment
FGF-5, FGF-18 [252]	Subretinal injection of adeno-associated virus vectors	Rescuing photoreceptors from apoptosis, mediating tyrosine kinase pathways	
Calpain inhibitor (1S-1-((((1S)-1-benzyl-3-cyclopropylamino-2,3-di-oxopropyl) amino) carbonyl)-3-methylbutyl)carbamic acid 5-methoxy-3-oxapentyl ester (SNJ-1945) [15]	Intraperitoneal (IP) injection	Restoring basal autophagy and suppressing photoreceptor death induced by N-methyl-N-nitrosourea (MNU)	
Methylene blue (MB) [253]	Dietary supplement	Decreasing photoreceptor cell survival and oxidative stress without correcting the energy deficit, normalizing the NAD + /NADH ratio and deactivating the mitochondrial stress response pathways, unfolding protein response and mitophagy	
Sustained production of GDNF by electrotransfer of GDNF-encoding plasmid (30 μg) in the RCS rat ciliary muscle[254]	Non-viral gene therapy	Warning against toxic effect of high dose of GDNF administration in the retina, neuroprotective effect in RCS with RP	
Cell therapy			
UCB-MSC	Subretinal injection	Secreting IL-6, bFGF, and BDNF, reducing functional deterioration in rodent model as well as photoreceptor degeneration	Unexpected pathology and cell differentiation into neurons were not observed [136]
BM-MSC	Subretinal, epiretinal and intravitreal injection	Stimulating and expressing bFGF, BDNF, and CNTF secretion in retinal layers [138, 139, 255], inhibiting photoreceptor apoptosis; thus slowing down retinal degeneration, integrating into ganglion cell layer to some extent [256]	In some cases, the injected cells had disappeared after transplantation [137, 139]
DPSC	Intravitreal injection	Secreting neurotrophic factors, Integrating into the photoreceptor layer, protecting the retinal morphology within two months	Differentiation potential of the transplanted cells needs to be investigated [141]

#### Periocular route

This delivery route administrates substances into periocular tissue, which is capable of local effects in periocular and/or intraocular tissues. The periocular path itself includes other different routes and has been investigated as a promising delivery way. Among these delivery ways, sub-tenon, subconjunctival, and suprachoroidal routes are usually chosen for delivery of the therapeutic agents to the posterior segment [51, 62]. Below, each method has been described briefly.

##### Sub-tenon

A sub-tenon injection is placed in an avascular area between the tenon’s capsule, a fibrous membrane, and sclera, providing an increased diffusion to the posterior segment [63]. The advantages of this way are the reduced therapeutic agent`s passage to the systemic circulation and the prolonged contact time with the sclera, and its disadvantages are pain, chemosis, subconjunctival haemorrhage, retrobulbar and/or orbital haemorrhage, optic nerve damage, retinal ischemia, orbital swelling, and rectus muscle dysfunction [51, 61, 63–65].

##### Subconjunctival

In the subconjunctival route, therapeutic agents are injected underneath the conjunctival that covers the sclera, thereby avoiding the conjunctival epithelial barrier. Therefore, not only direct access to the subconjunctival space is provided, but also therapeutic elements cross the sclera and choroid to reach the retina [51, 61, 66]. This route presents easier accessibility and reduced side effects, but it requires a higher injection volume with the same drug concentration compared to IVT. The limited concentration of agents in the retina limits the application of this pathway to the posterior segment [58].

##### Suprachoroidal

In suprachoroidal injections, drugs are administrated to the suprachoroidal space, a conceivable space between the sclera and the choroid. Minimizing the potential systemic side effects and increasing administered dose without IVT route-related side effects are the significant advantages of the suprachoroidal way [55]. So far, the highest achievable drug level via this route is 1 ml suspension or solution [61].

#### Sub-retinal

The sub-retinal injection is considered as another approach aiming at direct access to the PRs. This method has been mostly used for retinal gene, and cell therapies [67, 68] and all the procedures should be carried out in one step, as repeated treatments could lead to retinal detachment [57, 58]. The advantages of this approach are direct injection to the vicinity of the PRs, slight surgical invasion, and significant contact between the therapeutic elements and host cells [69]. For this purpose, the injection may be carried out via trans-corneal, trans-vitreal, or trans-scleral. Although the advantages and disadvantages of each method are discussed elsewhere [70], the argument in favour of each method remains among scientists. For example, trans-vitreal injection, which reaches the sub-retinal space vitreally, seems to offer greater visual monitoring of the procedure that is crucial from the surgical point of view [69]. While trans-scleral injection, which penetrates the choroid, can avoid breaching the retinal blood barrier, somewhat, yet may lead to blood vessel damage and flux of immune cells [71, 72]. Although this delivery route offers the possibility for avoiding the creation of retinal holes, and therefore risk of intravitreal extravasation of therapeutic product or retinal detachment, it can be technically more challenging and less frequently utilized. In theory, however, it might allow safer delivery to the subretinal space compared to the IVT route [55, 73].

In summary, to cross-compare the commonly used delivery routes, the least invasive and less direct routes of administration could provide significant benefit to patients, while avoiding the risks associated with a direct, invasive method such as intravitreal injection, including endophthalmitis [74]. However, local delivery is superior to systemic delivery in terms of significantly reducing dose and biodistribution, thus minimizing the potentially deleterious side-effects associated with the systemic application [69]. It should be noted that the diffusion across biological barriers in order to access the vitreous is significantly affected by formulations, dosages, sizes, charges, and structures of particles. To illustrate, large and hydrophilic compounds, particularly positively charged molecules, present a restricted diffusive movement [75]. As a result, successful clinical delivery to the posterior segment requires a fundamental consideration of the therapeutic elements, metabolism at the injection site, the nature of ocular barriers, and possible strategies to overcome these barriers.

### Carrier systems

The extensive research history exists behind the development of an appropriately suitable vehicle for therapeutic element delivery. Encapsulating elements into the suitable vehicles can cross the ocular barriers thus bringing the cargo release closer to the target. The different types of carrier systems, including nanoparticle-based drug delivery systems, have been studied extensively during the past decades for CNS and ocular delivery [57]. These technologies can improve the therapeutic efficiency, compliance, and safety of ocular therapeutic elements, administered via different routes. In the following sections, a summary of the common systems used for the delivery of pharmacological elements was outlined. However, carriers used for gene transfer such as viral and nonviral vectors as well as the substrates used for cell transplantation such as implants and scaffolds will be discussed in the related sections.

#### Nanoparticle-based delivery systems

Nanoparticle-mediated carrier systems have potential therapeutic effects due to their unique characteristics such as high biocompatibility, protecting the cargo, sustained release, improved bioavailability of the particle localization, and enhanced drug efficacy. Also, nanoparticles act as the active component of the therapy, such as Nanoceria which has shown promise for retinal disease [76–78].

Nanoparticles can protect delivery elements from systemic clearance by either encapsulating or modifying the drugs to the surface of the nanoparticles through adsorption or covalent bonding. They create a controlled release of the cargo substances to prolong the therapeutic window and enhance the bioavailability with improved stability and targeting effects. The process of nanoparticles crossing the barriers and the ratio of nanoparticles distributing into the respective tissues depend on the physicochemical properties of nanoparticles, in which the most crucial ones are size and surface properties [79].

Particle size has an impact on biodistribution, toxicity, targeting ability, as well as stability of the nanoparticles. Nanoparticles, a size ranging from 1 to 1000 nm, have been used for intravitreal drug delivery due to their ability to be injected with a small needle. For intravitreal injection, size has been investigated to play important roles in diffusing through the vitreous, crossing the inner limiting membrane, interacting with the cell layers, and uptake by the targeted cell. Although particles with smaller sizes can overcome the vitreous and subsequent barriers, they tend to have longer circulation time and a higher degree of toxicity [79, 80].

**Fig. 3:**
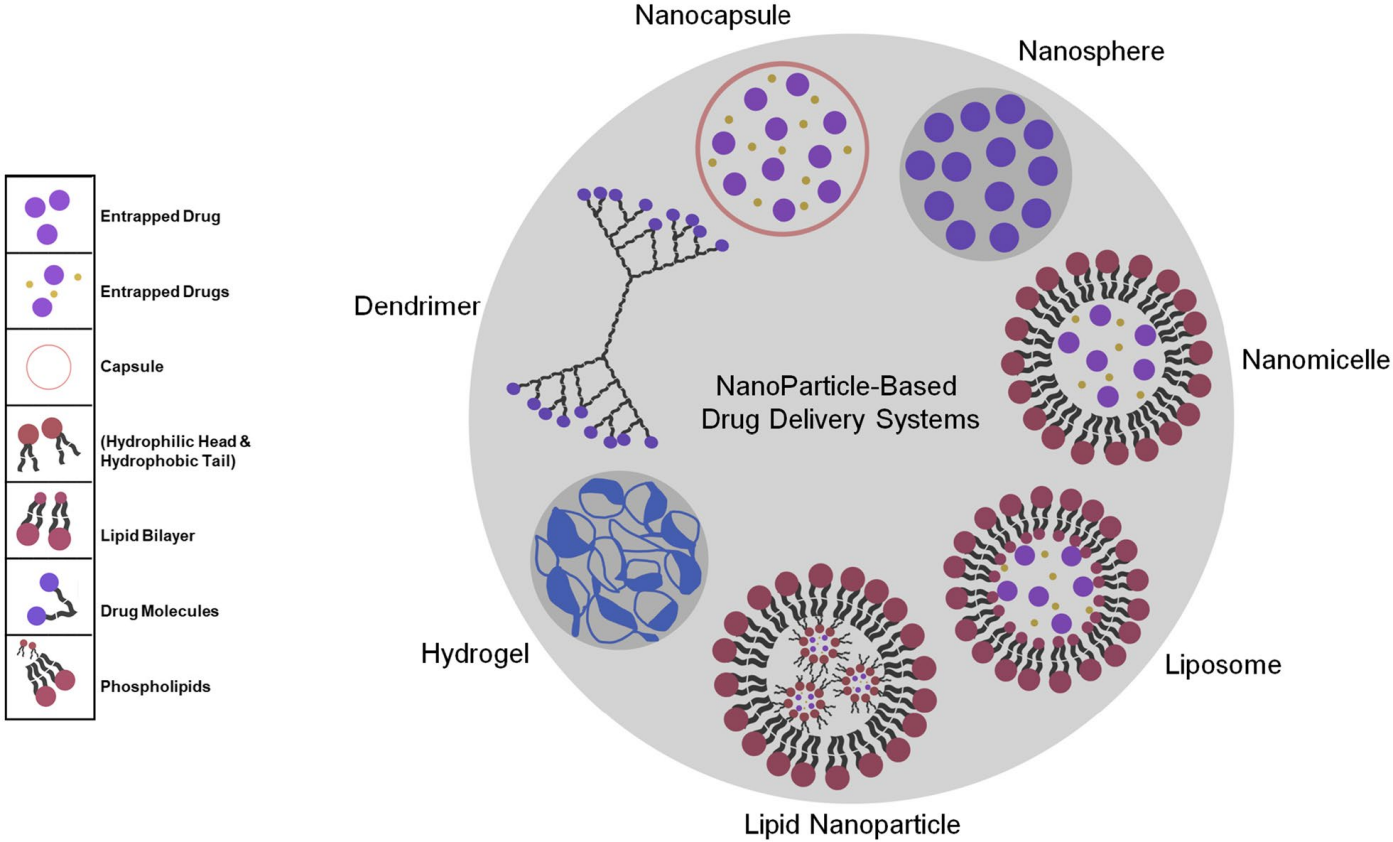
Nanoparticle-based drug delivery systems. Common carrier-based drug delivery systems of therapeutic nanoparticles: nanosphere, liposome, nanomicelle, nanocapsule, dendrimer, hydrogel, and lipid nanoparticle

The commonly used nanoparticle-based carrier systems and their different entrapping ways are outlined in the sections below (Fig. 3).

- Nanospheres are spherical nanovesicles with a size ranging from 10 to 200 nm and made of biodegradable polymers that protect the drug from degradation. In nanospheres, the drug is entrapped inside the polymer or attached to the surface of the particles and capable of sustained drug release [49, 76, 81].
- Nanocapsules, with a size ranging from 10 to 200 nm, are composed of a hydrophilic or lipophilic core or cavity surrounded by a polymeric membrane called the capsule. Nanocapsule, encapsulates relatively large amounts of drugs in the core, and present a more sustained release of the drug [76, 82].
- Liposomes are spherical amphipathic vesicles made of a naturally biocompatible phospholipid bilayer, resembling a cellular membrane that surrounds an aqueous core. Liposomes can entrap both hydrophobic (in the vesicle membrane) and hydrophilic (in the inner aqueous core) molecules. Liposomes, with their abilities in PR delivery of low molecular weight compounds and high molecular weight proteins, have been shown to afford for future drug delivery approaches. This delivery system offers advantages such as biocompatibility, biodegradability, low toxicity, self-assembling capability, and allowing repeated intraocular injections [49, 76, 83, 84]. Therefore, liposomes represent promising drug delivery systems (DDSs) and are widely used in preclinical studies for the treatment of retinal disease due to their high half-life, permitting the long term drug absorption as well as their capacity to significantly enhance pharmacokinetics and pharmacodynamics of any loaded drug compared to its free-circulating counterpart [85]. Surface-modified liposomes have been shown to enhance the efficiency of drug delivery. In this regard, liposome surface modification by the water-soluble cationic polymer poly-L-lysine (PLL) has been reported as a high biocompatible and low toxic cargo [86]. Evidence began to accumulate that a lack of consistency in the fabrication of liposomes and insufficient characterization of basic liposome features have a significant influence over in vivo outcomes. Thus, special attention should first be given to basic characteristics, including liposome constituents and resulting pharmacokinetics. Also, critical evaluations of the obtained results, the analytical techniques, and quantitative methods shall be taken into consideration to successfully develop the field [85].
- Nanomicelles are colloidal amphiphilic nanoparticles, with a hydrophilic shell and a hydrophobic core in which drugs are solubilized and encapsulated. Nanomicelles, as a novel ocular DDS allow enhanced peroneal residence, controlled sustained release of drug to the target area, and enhanced the bioavailability of the therapeutic agents with fewer side effects [76]. Nanomicelles are among one of the most widely studied nanoplatforms. It was reported that premature dissociation of the self–assembled multi-molecule polymer micelles could undermine their tissue-targeting capability and cause a burst release of the drug, potentially leading to systemic toxicity [87].
- Dendrimers are highly branched polymeric structures, which consist of a central core, branches, and terminal functional groups. Dendrimers are another DDS offering controllable nanoscale scaffolding and nanocontainer properties [88].

Whereas drug delivery vehicles including hydrogel and contact lenses would address residence time issues, developing novel carrier systems such as nanoparticles can alter lipophilicity and hydrophilicity of the prodrug to permeate throughout lipophilic barriers or dissolve in tear fluid, respectively [89]. Penetration enhancers such as quaternary ammonium compounds (cyclodextrins, chelating agents, crown ethers, bile acids, and bile salts) will also facilitate delivery of the therapeutic agents via temporarily modifying the membrane components [90]. Iontophoresis (penetration of ionized drug under the influence of low electric current) and sonophoresis (penetration of hydrophilic drugs and peptides at low (200–100 kHz) to high frequency (400–16000 kHz) ultrasound, respectively) have also been developed as non-invasive penetration enhancers [91]. A study has demonstrated that trans-scleral iontophoresis-assisted delivery of plasmid DNA will rescue PRs in the periphery as observed by green fluorescence protein (GFP) transfer [92].

#### Polymeric carrier systems

Polymeric biomaterials in natural and synthetic forms have been utilized as delivery substrates for various therapeutic elements in ocular regenerative approaches and have been extensively reviewed elsewhere [93, 94]. Polymeric nanoparticles can be made of various degradable biopolymers in which the active element is dissolved, entrapped, encapsulated or attached to the surface. Although they possess the features such as biodegradability, biocompatibility, ease and low cost of production, and the possibility to freeze-dry and reconstitute, as well as high constant stability, possible systemic toxic impacts from both polymer degradation products and residual organic solvents are the inevitable drawback of these systems [84].

### Delivery challenges

Delivery to the posterior segment faces significant challenges, including administration issues and drug targeting to the PRs considering the eye barriers. Thus, new strategies have been developed to impose these obstacles by prolonging drug residence time, increasing particle permeation through ocular barriers and inventing drug release inserts. Understanding the complexities associated with pharmacokinetics and pharmacodynamics would greatly aid further advances to the field [95]. For successful treatment, both drug formulation and delivery system must be accurately be chosen, especially regarding the route of administration.

Taken together, almost all treatments to deliver therapeutic agents to the PRs may lead to retinal detachment, haemorrhage and require patient compliance [96]. Thus, particular interest has sparked in sustained and targeted delivery using non-invasive nano carrier-based therapeutic delivery systems. However, translation of this system from bench to bed, which mainly depends on their scalability, dispersion safety and reproducibility, is somehow challenging [97]. Recent advance such as hydrogel carriers, microfluidics and bioprints have been developed to promote commercialisation [98].

Except for obstacles that particles face on their path to the PRs (where the disease manifest), each drug, specifically, has its own issues to be addressed. For example, protein and peptides including Bevacizumab [99], goat immunoglobulin G (IgG) [100], (Anti-VEGF agents) and Adalimumab (a monoclonal anti-TNFα antibody) [101] are a class of biopharmaceuticals, which have been used in PR degeneration. These biopharmaceuticals face major challenges, including their large size, lack of permeability and vulnerability to degradation [61]. Although common administration routes mentioned earlier can be implicated to deliver macromolecules, novel formulations have been developed, such as biodegradable polymeric micro/nanoparticles [102], delivery of positively charged proteins and peptides using negatively charged nanoparticles [100], sustained-release by in-situ gel formation [103], and delivery using encapsulated cell technology (ECT) [104]. Several delivery techniques for PR regeneration are in the preclinical stage, and some, like intravitreal implant of PLGA containing Brimonidine tartrate (Allergan) is completed (NCT00661479).

## Current approaches of photoreceptor regeneration

**Fig. 4:**
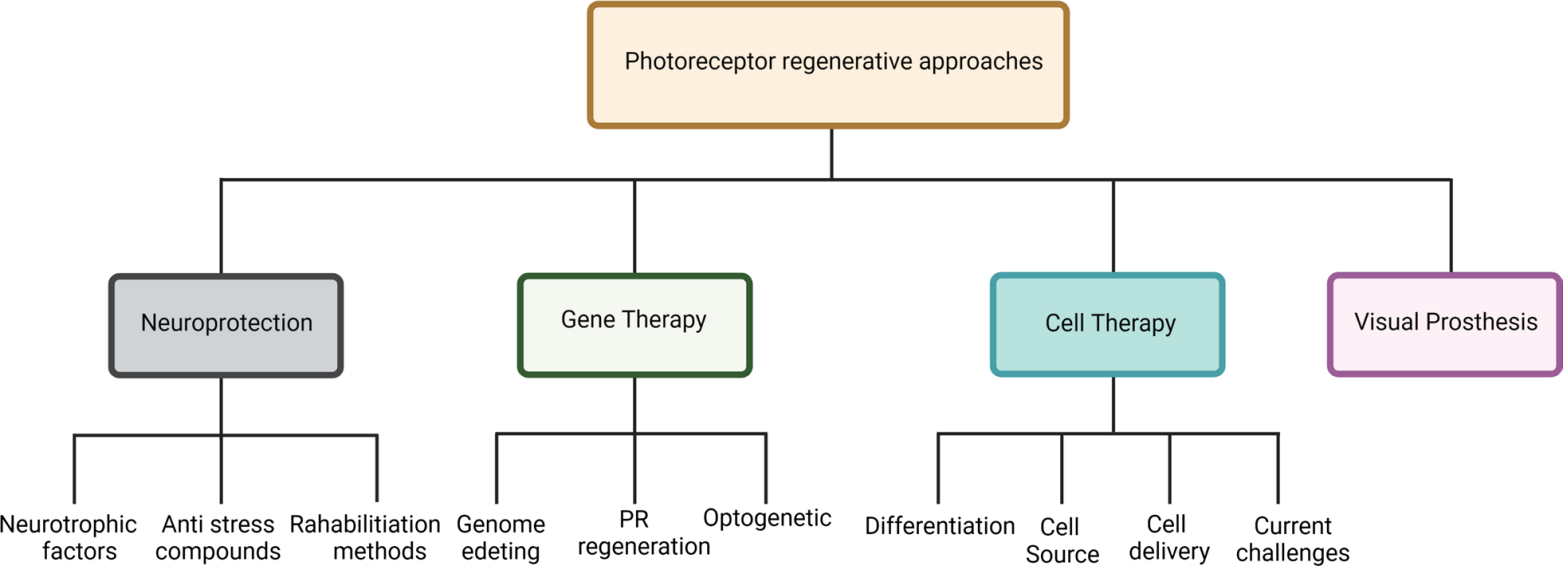
Different approaches for retinal regeneration. Current therapy methods have been categorized in neuroprotection, gene therapy, cell therapy, and visual prosthesis

In the following section of this review, we describe the innovations and recent developments in PR regeneration. Neuroprotection, gene therapy, cell-based therapies, and visual prosthesis are presently employed as the most opportune treatments in this growing research area (Fig. 4).

### Neuroprotective strategies

Neuroprotective strategies aim to preserve neuronal viability, particularly in the early stage of vision loss, offer the possibility of slowing the progression to severe visual impairment, and therefore providing hope to prevent blindness [105]. Neuroprotective treatments have been attempted for PR degeneration since as early as in 1950 [106, 107]. Table 1 has summarised the research programs and trials that have been carried out with neuroprotective strategies to preserve PR cells prior to end-stage progression; also we reported the state of progress of these trials towards clinical therapy.

The research history behind neuroprotection in retinitis pigmentosa (RP) is extensive and has included neurotrophic or anti-stress compounds, as well as stimulation of the signalling pathways by environmental stimuli, among various promising strategies, some of which have advanced to clinical studies. An in-depth discussion and analysis of the PR neurodegenerative mechanisms are beyond the scope of this review, however, the most common neuroprotective methods are outlined in the sections below.

#### Neurotrophic factors

Early attempts to retain neural viability include delivering endogenous or exogenous neurotrophic factors (NTFs) to neural retina tissues [108], which enhance PR cell survival through several neuroprotective interventions. NTFs consist of neurotrophins (NTs) generally, as well as related molecules, including nerve growth factor (NGF), brain-derived neurotrophic factor (BDNF), neurotrophin-3 (NT-3) [109], neurotrophin-4/5 (NT-4/5) [110], glial-derived neurotrophic factor (GDNF) (neurturin, artemin, persephin), the serine protease inhibitor (srpin) family, pigment epithelium-derived factor (PEDF), neuropoietic cytokines (ciliary neurotrophic factor (CNTF)), insulin-like growth factors, and transforming growth factors (TGF) [111, 112]. These neurotrophins, which can be released by glial cells, including microglia and macroglia [113, 114], are found in photoreceptor segments [115]. Gene delivery [116, 117] or intraocular injection [118] of the neuropoietic cytokine CNTF rescues PR cells in several types of genetic degeneration or from the damaging effects of constant light [119]. However, the clinical efficacy of CNTF for PR degenerations may be questionable [120] since CNTF transgene expression delays PR cells degeneration in small animal models, but may not rescue normal retinal function of the preserved cells [121]. Continuous BDNF expression in double transgenic mice protected PRs cells significantly, whereas intravitreous injections of BDNF in mouse models showed minor survival-promoting activity [122, 123]. PEDF is another neuroprotective factor expressed by RPE cells and externalized into the surrounding interphotoreceptor matrix where it in turn could function as an anti-inflammatory and neurotrophic element providing PR protection [124, 125]. Moreover, recent studies have demonstrated that these activities are conferred by PEDF peptides (amino acids that span between positions 98–114 of the human PEDF sequence), which affords cone neuroprotection of a mouse model upon a single intravitreal injection [126, 127].

Despite the undisputed benefits of neurotrophic factors mentioned above for the reduction of PR cell death, there is still extensive discussion regarding the risks associated with others like vascular endothelial growth factor (VEGF). Clinical data have implicated the potential damage to the retinal neurons in patients treated with frequent administration of anti-VEGF injections [128]. In fact, monthly intravitreal injections of VEGF have promoted PR regeneration in rd1 mouse model by stimulating the proliferation of the retinal progenitor cells (RPCs) [129]. Therefore, the possibility that exogenous VEGF promotes PR survival conflicts with the advantages of VEGF suppression in the treatment of retinal and choroidal vascular diseases [130].

Although, the neuroprotective effect of these neurotrophic factors is limited by their relatively short half-life in the vitreous (e.g., 1.5 min for CNTF [131]), several attempts have been made to develop delivery systems that could increase the availability of these agents for a longer period. Some delivery alternatives which bring neurotrophic factors closer to the clinic are: the use of cell-based delivery methods such as encapsulated cell intraocular implants (CNTF release from encapsulated cells) [104, 132], gene-therapeutic methods such as intravitreal injection of GDNF-secreting mouse embryonic stem cells in the transgenic S334ter rat [133], repeated microinjections into the suprachoroidal space [134], adeno-associated virus (AAV)-mediated delivery of CNTF [117]. Also, the combination of growth factors has been successfully implemented to enhance neurotrophic potential compared to the delivery of a single growth factor with improvement in the overall efficacy of neuroprotection [135].

Cells can also deliver neuroprotective factors to the target area as a function of their own secretome. Numerous studies have demonstrated that mesenchymal stem cells (MSCs) provide protection effect via a variety of growth factors, as well as immuno- or apoptotic modulators. Several reports have shown that MSCs from different sources possess neurogenic and neuroprotective capabilities capable of protecting PRs from degeneration. Human umbilical cord blood MSCs, injected into the subretinal space of RCS rats, significantly reduced the degree of PR degeneration and this was attributed to secretion of neurotrophic factors, such as FGF2 and BDNF [136]. Transplantation of bone marrow mesenchymal stem cells (BM-MSCs) in subretinal [137], epiretinal [138], or comparatively subretinal/intravitreal spaces [139] increased PR cell survival and rescued retinal function in the rat model of retinal dystrophy.

Recently, Usategui-Martín et al., evaluated the neuroprotective potential of the hBM-MSCs secretome using the ex vivo model of spontaneous neuroretinal degeneration and identified paracrine factors secreted in a co-culture system. The expression of several proteins with antioxidant, anti-apoptotic, or anti-inflammatory activity were detected in the neuroretina co-cultured with h-BM-MSCs. They also reported that hBM-MSCs expressed a variety of factors which could protect against PR degeneration, including GDNF, BDNF, PDGF, and CNTF [140]. The in vitro evidence showed that secretion of cytokines, neurotrophic factors, and anti-inflammatory paracrine factors by hBM-MSCs likely plays a key role in the therapeutic effect.

Dental pulp stem cells are another source of MSCs revealed to overcome PR cell death and possess increased neurogenic and neuroprotective capabilities as well as functional preservation after being injected into the intravitreal space in a rat model of NaIO3- induced retinal degeneration. This could be explained by the heightened expression of NTFs [141].

Another cell type of interest is the RPC, which will also be discussed later. These are multipotent cells of neural lineage, either derived directly from fetal retinal tissue or, alternatively, differentiated from pluripotent cell types using defined protocols. RPCs have been viewed as a candidate cell type for use in photoreceptor replacement applications based on their pivotal role in generating photoreceptors during retinal development. Animal studies confirmed integration of allogeneic RPCs into the retina, which was associated with functional improvement [142]. These cells can also be derived from prenatal human donor tissue [143]. Interestingly, the cells have shown utility as a method of retinal neuroprotection in retinitis pigmentosa, based on ongoing clinical studies (Table 1).

There is growing evidence that neurotrophic protection might be achieved by material transfer, suggesting a powerful new mechanism to improve dysfunctional and degenerating PRs [144]. Transplantation of reporter-labelled post-mitotic photoreceptor precursor cells into the subretinal space followed by improvement of visual function was understood to be due to donor and host PRs engaging in transfer of cellular material, either as RNA and/or proteins. The evidence presented indicated that donor PRs might not structurally integrate into the retinal tissue but instead exchange intracellular contents with host retinal cells. The in vivo material exchange, including RNA and/or protein, has been detected between host and donor cells, however, actual fusion between donor and host cells has not been demonstrated [145]. Therefore, in addition to donor cell integration, which has been reported previously, material exchange may represent an additional mechanism by which vision can be rescued via cell therapy [146].

Topical administration of αlpha-2 adrenergic agonist brimonidine (BRM) has potent neuroprotective effects against phototoxicity [147] and maintained the photoreceptors in animal model of diabetes [148]. Vidal-Sanz et al. has extensively studied the neuroprotective effects of BRM in retinal degeneration via administration route [149, 150].

In addition, microglia activation as a trademark side factor of retinal degeneration is also an important issue, which is broadly investigated by differet research studies and the controlling mechanisms such as anti-inflammatory components might be applicable. The reports have been revealed that Minocycline, a semi-synthetic tetracycline analog, or 2, 2′-aminophenyl indole (2AI) showed a potent immunomodulatory as well as neuroprotective effects via reducing nitric oxide production or pro-apoptotic gene expression [151–153].

Extracellular vesicles (EV), especially exosomes, provide a means by which intercellular material transfer might occur, and therefore represent another potential source of neurotrophic agents for PR regeneration. The EVs, as a mechanism of messaging and transfer of subcellular materials, deliver protein and miRNA cargoes and can reportedly play a therapeutic role in retinal regeneration, mainly by a miRNA exchange mechanism [154, 155]. EVs consist of heterogeneous sub-micron populations, including exosomes (30–110 nm), ectosomes, endosomesoncosomes, microvesicles (200–500 nm), apoptotic bodies (500–1000 nm) [156]. The heterogeneous nature of EV populations, the ability of EVs to reach inner retinal layers, as well as the reduced risk of donor cell malfunction post-injection, would be potential advantages of a cell-free, EV-based neurodegenerative ocular disease therapy.

Intravitreal injected EVs, derived from MSCs, successfully delivered their cargoes to the inner retinal layers and partially prevented axonal loss and degeneration following mechanical [157] or optical [158] injuries. Exosomes from RPE, used in combination with mouse retinal explants exposed to oxidative stress, demonstrated a potential mechanism for PR neuroprotection based on secretion of αB crystalline (a chaperone protein) that was taken up by photoreceptor cells [159].

Nevertheless, EVs can also play a neurotoxic role by transferring potentially deleterious exosomal miRNAs or lipids into the recipient neural cell or, similarly, spreading toxic proteins through exosomes to neighbouring neural cells [160]. For instance, EV activity was regulated via inhibition of Poly ADP ribose polymerase (PARP) activity, which is involved in PR degeneration. PARP inhibition protected rod photoreceptors in a PDE6b mutation model [161].

In conclusion, much research remains to be done to reveal the full potential of therapeutic intervention with EVs in PR neuroprotection.

#### Anti-stress compounds

The following sections provide a detailed explanation of the neuroprotective compounds, including antioxidants, synthetic bile acid, steroid hormones, and dopamine, by reviewing their history, mechanism of action, and further consideration in bringing the compound closer to the clinic (Table 1).

##### Antioxidants

The high metabolic rate of retina increases the chance of the cells’ reactive oxygen species (ROS) exposure as a byproduct of the redox-regulating system. The application of antioxidants, including vitamin A/E/C, docosahexaenoic acid (DHA) and glutathione (GSH) has been a focus of investigations as a mean of PR neuroprotection via increasing the resistance of neurons to oxidative stress [257]. Beginning in the late 1960s, Berson et al. conducted several studies and clinical trials on retinitis pigmentosa patients who took vitamin A alone or in combination with other products [258–265]. They reported that protective effects are under the influence of experimental designs such as age [260, 266], gene mutation [267] and type of supplementation [268]. For instance, vitamin A was found to be relatively effective in clinical trials comparing vitamin A alone or in combination with vitamin E, DHA, or lutein [261, 263, 269]. In another study, declination of the visual field was reduced in patients who had an omega-3 rich diet and were taking vitamin A [270]. As for genetic mutations, Jin [271] used patient-derived induced pluripotent stem cells (iPSCs) to construct patient-specific rod cells. These cells with specific mutations respond differently to vitamin E than had been observed in clinical trials, most likely due to the different underlying mutations. Similarly, differences among species would lead to inconsistent responses in different animal models. Komeima and coworkers [272, 273] studied a mixture of antioxidant treatments with α-tocopherol, ascorbic acid, Mn (III) tetrakis (4-benzoic acid) porphyrin, and α-lipoic acid on rd1 mouse model of RP, rd10/rd10 mice (a model of more slowly progressive recessive RP) and Q344ter mice (a model of rapidly progressive dominant RP). Treatment with the antioxidant mixture resulted in moderate preservation of cone cell receptor in rapidly progressive RP regardless of the reason behind the rod cell death. Whereas, animal models with slowly progressive RP interestingly showed prolonged rod survival suggesting that rod cell death due to the oxidative stress may contribute in more slowly progressive diseases. In another study, the synergic effect of a combination of lutein, zeaxanthin, alpha lipoic acid and reduced l-glutathione antioxidants were reported on the rescue of rd1 PRs whereas individual antioxidants had no significant survival effect [274]. Antioxidants can be taken orally, however, variables such as dosage, age, and genetic mutations are not established yet.

##### Synthetic bile acid

Taurine-conjugated derivative tauroursodeoxycholic acid (TUDCA) was first introduced as a potent antioxidant by a research group at the University of Alicante [275]. Significant quantities of TUDCA could be found in the bile acid of hibernating black bears; also, this compound has been synthetically available since 1954. Despite the positive results obtained from the application of TUDCA on PR regeneration through protein folding and trafficking enhancement and/or ER and oxidative stress reduction [169], appropriate dosing and mechanism of actions of this compound require further extensive in-vivo trails. As such, much uncertainty still exists about the mixed effects were observed in animal models treated with TUDCA. For instance, in rd1 mice suffering from progressive retinal disease, daily intraperitoneal injection of TUDCA reduced PR degeneration [162] whereas, this dosing was not sufficient to stop PR cell death for rd1 and rd16 mice in another study [166]. Hence, to deliver the high dosage of the drug, Fernandez-Sanchez et al. have assessed an intravitreal controlled-delivery TUDCA encapsulated poly D-lactic-co-glycolic acid (PLGA) microspheres in a rat model of RP [163]. Furthermore, TUDCA significantly improved PR cell survival after retinal detachment by reducing oxidative stress compared to controls, though TUDCA did not affect ER stress in this retinal detachment model [170]. TUDCA, likewise, preserves the network of blood vessels and prevents the reorganization of the retina observed in the late stage of retinal injury in a mouse model of RP [276]. Taken together, TUDCA, as a FDA-approved compound is a promising neuroprotective factor, which has already begun translating into the clinic (Table 1).

##### Steroid hormones

Steroid hormones such as progesterone are synthesized in peripheral tissues as well as in the central nervous system (CNS). Their neuroprotective effects in CNS have been widely described [277]. As with the brain, the presence of hormone steroid receptors and steroid enzymes make retina a site of steroid production. Neuroprotective effects of progesterone exploit on multiple pathways, like (a) decreasing oxidative stress; (b) reducing inflammatory cytokine levels (IL-1β, TNF-α, IL-6, COX-2, and the p65 NF-κB subunit) [193]. (c) decreasing cellular apoptosis via increasing anti-apoptotic proteins (Bcl-2, Bcl-xL), and reducing pro-apoptotic proteins (Bax, Bad, caspase-3) [278], as well as (d) up-regulating the inhibitory neurotransmitter γ aminobutyric acid (GABA) [279]. The aforementioned mechanisms are known to be effective in PR cell survival [280]. Sánchez-Vallejo et al. reported that oral administration of progesterone delays PR cell death in a rd1 mice model of RP. In this study, progesterone acted on multiple levels like reducing retinal glutamate concentrations at PN15 and PN17 and increasing oxidized glutathione retinal concentrations [281]. In another work, PR cells survival was improved up to 70% in two different diseased mouse retinas; the light damage model and the Pde6brd10 model [282]. However, it appears that the effectiveness of the treatment with neurosteroids significantly depends on synthetic details (chemical structure and receptors they bind to), optimal dose, timing, and tapering of dose [283]. Being safe and FDA-approved can lead toward the therapeutic application of the treatment with steroid hormones in the nearest future [284]. However, successfully completed the clinical phases of this treatment require overcoming the mentioned experimental design limitations.

##### Dopamine

Previous studies have identified potential roles for dopamine in multiple retinal functions [285, 286]. Acting via dopamine D2-like receptors such as D4Rs in PR cells, DA modulates the production of cyclic adenosine monophosphate (cAMP), downregulating melatonin synthesis [287], and regulating the cell to cell coupling among horizontal cells depending on the phase of the light cycle [288]. Dopamine has also been revealed to act as a chemical messenger in trophic functions of the retina [289, 290]. Although dopamine has been reported to have either protective or toxic roles on neurons (most likely due to the malfunctioning mechanisms regulating dopamine homeostasis) [291], yet it is one of the few neuroprotective factors applied in humans. Levodopa, also known as L-Dopa and l-3,4-dihydroxyphenylalanine, has been effective on the patient with AMD [199, 203], contrast sensitivity [292], visual alterations in de novo Parkinson’s disease [293], ischemic and traumatic optic neuropathies [294–296], and rhegmatogenous retinal detachment [289]. However, to translate treatments targeting dopamine to the clinic, systemic side effects observed in Parkinson’s patients, like insomnia, gastrointestinal symptoms, motor side effects and hallucinations need to be considered [297–299]. Moreover, delivery methods of the drug to the eye [198], type of the drug (L-DOPA, dopamine agonists, dopamine transporter inhibitor) [297, 298, 300] and optimal dose for the retina disease are fundamental considerations, which must be determined to achieve a highly efficient dopamine based neuroprotective treatment.

#### Rehabilitation methods

The protective effects of rehabilitation approaches, including exercise and electrical stimulation, have also been a focus of recent investigations as a promising modality of PR regeneration. The rehabilitation approaches appear to act via multiple pathways such as modulation of BDNF and VEGF and/or mediation of BDNF/TrkB signal transduction. For instance, exercise increased retinal BDNF protein levels by 20% in wild-type BALB/c mice, which were exercised on a treadmill then exposed to toxic bright light; therefore, PR regeneration was implicated by BDNF signalling [242]. Some reports revealed the involvement of FNDC5/irisin pathway in the upstream of BDNF following exercise [301]. Another research has been performed in this field indicating that a similar pattern of retinal leukemia inhibitory factor (LIF) gene expression was induced by aerobic exercise preconditioning and protect PRs against light-induced retinal degeneration (LIRD) [302]. Moreover, TrkB activation was found to mediate exercise’s preservation of the retina in rd10 mouse model of RP that was voluntary active [241].

Electrical stimulation is also considered as a rehabilitation method providing neuroprotection in different animal models of retinal degeneration [245, 247]. Electrical stimulation, which can be applied via inserting electrodes in the subretinal space or on the cornea, preserves retinal function through inducing growth factors [244], down-regulating IL-1β, TNFα, and Bax, and up-regulating Bcl-2 [246]. For instance, subretinal electrical stimulation (SES) from a microphotodiode array (MPA) promotes the signal transmission through the retinal network and consequently preserves PRs in RCS rats [248]. Furthermore, transcorneal electrical stimulation (TCES) was found to delay PR degeneration by activating the intrinsic survival system, such as selectively up-regulating Bcl-2 and CNTF in Müller cells [246]. A clinical trial has also been carried out on human suffering from decreased vision due to multiple sclerosis. The primary outcome showed partial vision improvement after eight weeks of treatment with transcorneal electrical stimulation (NCT02019927).

Besides the mentioned benefits of rehabilitation methods to the PR neuroprotection, optimal stimulation parameters, or exercise regime (speed, duration, and the type of exercise) need to be addressed prior to its implication into the clinic.

### Gene therapy

The eye is a unique organ for therapeutic trials based on gene transfer owing to its anatomically accessibility for surgical injection and its immune-privileged status due to the presence blood-retina barrier. Also, its small size requires a low gene/vector dose to achieve a therapeutic response [303, 304]. Gene therapy has an advantage over traditional pharmacological approaches because of its long period effect without the need for repeated interventions [304].

As an efficient treatment focus on the retina is an especially appropriate organ for restorative obstructions. PR dysfunction is an ideal translational model for the development of gene replacement therapies targeting PRs.

#### Genome editing strategies: carriers and modalities

Gene therapy as a strategy for disease treatment requires a safe and effective gene carrier because nucleic acids don’t pass across lipid bilayer cell membrane. Finding a suitable vector for delivering therapeutic genes without causing cell injury, oncogenic mutation, or inflammation makes gene therapy a promising modality compared to conventional methods. IRDs are permanent blinding situations due to mutations in genes expressed in PRs and RPE [305]. Present clinical trials on gene therapy in the eye contain the delivery of exogenous genetic information into the cell with inherited genetic defects. Carrier used to transfer genetic material, introduce the gene of interest into cells [306]. This delivery can befall via viral or non-viral vectors. Each of these systems has different strengths and weaknesses, and the selection of the vector used mainly depends on the application.[307]. The perfect vector would deliver an adequate size of foreign genes into the target cell, thereby allowing for the expression of the gene and being non-immunogenic and safe.

##### Viral vector

In the past decade, the application of viral vectors in gene therapy has been increased notwithstanding this technique yields high technical demands and an increased risk of virus-associated toxicity. However, significant improvements have been introduced in the viral vectors replication component to make them safe [308]. During induction, the viral vectors transfer DNA to the host without appealing an immune response. Though viral vectors are presently the best option as a delivery system, the optimal method for delivering genes to the RPEs and PRs remains to be improved in order to rise transduction efficacy and reduce iatrogenic conditions [309].

There are two classes of viral vectors: integrating and non-integrating viral vectors. It is the ability of retroviral, lentiviral, and adeno-associated viral (AAV) vectors incorporate into the genome; whereas the non-integrating vector (e.g., adenoviral vector) is preserved in the nucleus without integrating into the chromosomal DNA, so the foreign gene is lost throughout cell division, and the expression is transient [310].

###### Retroviruses

Retroviruses are RNA viruses that use RNA as a target to transcribe into double-stranded DNA. This intermediated DNA is then fused into the host DNA, so machinery of the host cell produces all necessary viral components. Since the viral genome is able to integrate into the host DNA, all modifications will be transferred to all cells derived from the transfected cells [311]. Apart from the large-scale usage of retroviruses-mediated transfer of genes, the major limitations of retroviral vectors are low vector titter, and low transfection efficiency that confirmed in vitro experiments. Moreover, retroviruses are disabled to transduce gene into non-dividing cells, and target only proliferating cells [312].

In 1987, Connie Cepko evaluated the intraocular transfer of retroviral vectors to the rodent RPCs with reporter genes. Though it was not a therapeutic experiment, this study was the first report of in vivo gene delivery to the retina using retroviral vectors for delivery [313]. In previous lectures, some physical approaches have been exploited to recover the efficiency of retrovirus-mediated gene transfer owing to little retroviral titter.

Combined with the safety of gene transfer, it shows that ultrasound-assisted gene delivery has broad prospects as a new method to improve the efficiency of retroviral gene delivery. De-Kuang Hwang et al. demonstrated the capacity of ultrasound standing waves to improve retroviral transduction into retinal stem cells (RSCs). Their study was designed to use acoustic waves to improve the efficacy of gene delivery for RSCs [314].

###### Lentiviruses

Lentiviruses are known as a superior group of retroviruses that can be used to infect both proliferating and quiescent cells. Lentiviral transfer systems efficiently maintain expression and effective transfer without systemic inflammation [315]. Lentiviral vector gene–carrying capacity is between that of adenoviruses and AAVs, with a maximum cargo of nearly 8 to 9 kb [306].

Thus, it seems that lentiviral vectors are not suitable for use in post-mitotic tissue such as retina because of their ability to infect dividing cells. Yao and colleagues applied the subretinal injection lentiviral vectors containing neuraminidase and observed that the interphotoreceptor structure had been changed [316]. Besides, Gruter et al. have demonstrated that the lentiviral vector can transduce PRs in the young rodent retina, whereas it is poorly efficient for PRs of adult animals [317]. In conclusion, lentiviral vectors may be beneficial for the treatment of PR disorders by facilitating the transmission of secreted factors, such as neurotrophic or antiapoptotic factors, to RPE [318, 319].

###### Adenoviruses

The target gene is not incorporated into the host genome by the adenovirus. In other word, the adenoviral genome leftovers in the nucleus as an episomal component after the contamination of the host cell [320]. Adenoviruses-mediated transfer of gene has some advantages including; easy purification, and the high-efficiency rate of host cell infection, dividing or quiescent cells. These advantages make adenoviral vectors the most frequent vehicle for direct in vivo gene transfer. The disadvantages of these vectors are the episomal nature of viral genes, and they are also expressed immune response factors to the transduced cells [321].

In 1996, Bennett et al. delivered a cDNA copy of the phosphodiesterase β subunit to PRs in the rd1 mouse model using adenoviral vectors. They found that PR degeneration was delayed by 6 weeks [322].

###### Adeno-associated viruses

AAVs have high transduction proficiency and cell type specificity as well as low immunogenicity, making them an attractive tool for gene therapy. AAVs have been demonstrated that have no known pathogenicity, target non-proliferating cells, low immunogenicity, and may have distinct genome insertion sites [323]. These vectors have a smaller packaging capacity (4.7 kb) than lentivirus (8 kb), meaning they can’t carry gene coding exceeded than 5 kb. Scientists are also endeavoring to split transgenes or generate minigenes between AAV vectors, to overcome the size limitations of AAV. Over the past decade, AAV vectors most widely used for retinal gene transfer. For overcoming the small size of AAV, the dual AAV vectors each of which contains half of a large transgene expression cassette have been engineered. However, dual AAV vector PR transduction efficiency is currently lower than with a single AAV vector [324]. Because of the low integration frequency, low immunogenicity, and non-pathogenicity potential of AAV, it is the best way to improve safety profile in comparison to other viral vectors [325, 326]. Recently, thirteen wild-type AAV serotypes (AAV1–AAV13) have been isolated, and AAV2-based vectors have been reported to have a good affinity for PRs and the RPE [327]. Non-viral gene delivery methods, in addition to viral-mediated gene replacement therapies, guarantee the continuous fine-tuned expression of secreted protein therapeutics that can be adapted to the evolving stage of the course of the disease and can address more common non-genetic retinal diseases.

##### Non-viral vector

Although significant progress in gene therapy has been made in studies on the application of viral vectors, they cause immunogenicity and pathogenicity. More recently, the application of non-viral gene therapy has been developed for the transduction of genes because of its less immunogenic and expensive than viral approaches [328]. Non-viral such as naked DNA, nanoparticles, and liposomes can be used to deliver DNA of unlimited size [329], which is explained more here.

###### Liposomes-based methods

Liposomes as the self-assembled structure are explained above, have been tried in order to gene delivery to the eye [330, 331], however, the efficiency concerning viral vectors is lower, and the effects are transient [332]. Asteriti and colleagues injected lipid nanovesicles into the vitreous body of the eye and observed increased sensitivity of rod photoreceptors to light [333]. However, the use of liposomes to target PRs gave unpromising results [334]. Besides, Kachi et al. delivered liposomes to the subretinal space and found that RPE cells were transfected, but PR transfection was not observed [335]. Moreover, liposomes have many advantages for gene transfer, but they tend to aggregate following administration which can interfere with vision as well as result in retinal toxicity [336]. In conclusion, it seems that liposome-mediated gene delivery is not a suitable approach for promising results in the therapeutic use of IRDs, at least for the moment.

###### Naked DNA plasmid

Gene transfer with naked DNA plasmid exposed to intracellular nucleases would result in DNA degradation [309]. Electroporation with naked plasmid DNA improves DNA cell entry into ocular cells because it facilitates cell permeabilizations for enhancement of gene delivery into the cells [337]. In the eye, plasmid DNA electroporation was applied to transduce retinal cells. Efficient gene transfer to the neuroretina [338], RPE, and PRs by electrotransfection was achieved following subretinal injection of naked plasmids [339, 340]. Iontophoresis has been applied for transferring plasmids through sclera and express transgenes in the PR layer of adult normal and newborn rd1 mice [341]. In general, it is essential for naked plasmid DNA to protect against endonuclease degradation in the cytosol, and the approaches that would delay of endosomal escape might be applicable in gene therapy.

###### Nanoparticle-based methods

Nanoparticles (NPs) for gene therapy are categorized broadly into three groups including; (1) metal NPs, (2) lipid NPs, and (3) polymer NPs. For enhancing their efficiency in gene delivery, they should be uptake by cells, escape from endosomes and deliver the DNA plasmid to the nucleus for gene expression [342]. Recently, NPs have developed as gene nanocarriers to the retina. Kim et al. applied the intravitreal injection of gold NPs to the eye and found that they were non-toxic to the retina [343]. Rds gene encodes retinal disease slow (RDS) protein which is known as peripherin/rds. The subretinal injection of NPs carrying Rds plasmid induces Rds gene expression in PR cells [344, 345]. Mitra and colleagues injected RHO DNA NPs into the sub-retinal space of the Rho^P23H/P23H^ knock-in mouse eyes and observed partial improvement in structural and functional recovery of rod cells [346]. Perfection in the mouse model of RP was observed after NP-mediated genomic DNA transfer [347]. Finally, despite the greatest potential benefits of non-viral retinal gene therapy, the issue of transient expression is an obstacle not yet overcome in this delivery system. Future developments may include new engineering to overcome their present limitations to long-term expression associated with non-viral gene delivery.

###### Gene silencing (siRNA)

One of the achievements in gene therapy is the inhibition or reaction of damaging effects that can be permitted by targeted inhibition of gene interested expression. Inhibition of gene expression has been applied in some retinal diseases by antisense oligonucleotides, aptamers, and siRNAs [348, 349]. siRNA is a potent inhibitor for gene knockdown which sequence-specific gene loss of function has been established in some tissues in vitro and in vivo [350]. The sequence specificity of siRNA associated with a local direction in the temperately isolated confinement of the eye provides an ideal setting for eye-specific gene interruption [351]. Owing to the tiny half-life of siRNA molecules, a daily reversible and dosage-variable treatment routine by non-invasive transfer is necessary [352].

Because of immune-privileged and surrounded properties of the eye, it is a suitable target for siRNA [350]. siRNA, as a therapeutic approach, has several key advantages containing sequence-specific targeting of almost any molecular target, access to currently ‘‘undruggable’’ targets, and relatively easy design at low cost. Despite these gains, the delivery of siRNA to the posterior section of the eye, such as the RPE segment, is not trivial for numerous reasons that assume are too large, hydrophilic, and negatively charged and hence cannot across cell membrane alone. This molecule degrades with a nuclease enzyme [353]. Besides, siRNA can also stimulate the immune system through the Toll-like receptor pathway [354, 355]. siRNAs have been tested in the pathogenesis of glaucoma, RP and neovascular eye diseases such as AMD, diabetic retinopathy (DR), choroidal neovascularisation (CNV) for animal models, and clinical trials have been shown with some of them [356].

Reich et al. applied subretinal delivery of siRNA to loss of function of VEGF in vivo in mice. The same study was performed on primates. In both cases, siRNA targeting VEGF considerably decreases the amount of neovascularization in the model of CNV [357, 358]. In the additional paper, siRNAs targeting either VEGF, VEGFR-1, VEGFR-2, or a mix of the three were displayed to reduce neovascularisation, herpes simplex virus-induced angiogenesis as well as against lesions of stromal keratitis [324].

However, delivery strategies that defend the siRNAs from degradation and are suitable for prolonging the gene silencing activity would help to improve the efficacy of RNAi-based therapies for ocular pathologies. Incorporating the siRNA into a carrier with chemical modification can relatively overcome the aforementioned limitations. The applicability of the carrier varies from one tissue to another, so careful in vitro testing in physiologically relevant tissue models is required to select the ideal siRNA delivery system for the target of interest [350].

###### CRISPR/CAS9-mediated genome editing in the retina

Clustered Regularly Interspaced Short Palindromic Repeat (CRISPR)/ CRISPR-associated protein 9 (Cas9) system is a more powerful tool to edit the genome. CRISPR/Cas9 system was found in bacteria and archaea as a protective method of evading viruses, and other foreign nucleic acids [359–361]. CRISPR/Cas9 technique, as an accurate efficient, and powerful means of multiplex gene editing, requires a short guide RNA (sgRNA), which makes it more accessible than other techniques in disease modelling and gene therapy exploration [362–364]. CRISPR/Cas9 system consists of two main components. The first component of the CRISPR/Cas 9 is an RNA molecule known as the guide RNA (gRNA), which finds and binds to the sequence of DNA that must be edited. The second component of the CRISPR-Cas9 system is a non-specific CRISPR-associated endonuclease Cas9 which is responsible for locating and cleaving target DNA [365]. In CRISPR/Cas9 technologies, double-strand breaks (DSBs) form target loci, as a consequence of the direction of single-guide RNA (sgRNA) to Cas9. Homology-directed repair (HDR) or non-homologous end joining (NHEJ) processes have been used for DSBs editing [366–368]. Due to the absence of a DNA template for the NHEJ repair pathway, this process is random and hence extremely error-prone, and can introduce additional insertions and deletions (indel) in DSBs. So, NHEJ has been extensively used for gene deletion [369].

CRISPR/Cas9-mediated gene therapy is known as a powerful multiplex gene editing modality owing to the ability to introduce or knock out multiple genes. Benjamin et al. applied a subretinal injection of gRNA/Cas9 plasmid in rat models of autosomal dominant RP. They found that the CRISPR/Cas9 system could cut off the RHO gene carrying the dominant S334ter mutation, leading to prevent retinal degeneration, and consequently, an improved visual function [370]. Moreover, Lattela et al. applied CRISPR/Cas9 technology for editing the human RHO gene in a mouse model with autosomal dominant RP. Their results confirmed that the effectiveness of the CRISPR/Cas9 system as a valuable and powerful method for gene-editing in PRs [371]. The knockout of the Nrl gene, as a crucial player in rod cell fate, in adult rods leads to the loss of rod function. Yu et al. applied (AAV)-mediated CRISPR/Cas9 delivery to post-mitotic photoreceptors. The results showed that CRISPR/Cas9-mediated Nrl disruption improves rod survival, consequently preventing secondary cone degeneration [372]. Generally, CRISPR/Cas9 technology seems to be a powerful tool to edit the genome specifically and effectively in retinal degeneration. However, off-target effect can alter the function of a gene and may result in genomic instability, which is a major challenge in bringing this technique forward. Although targeting specificity of Cas9 is firmly regulated by sgRNA, its off-target cleavage potential activity could occur on DNA thereby leading to three or five base pair mismaches. This disadvantage prevents its prospective in the clinical procedure [373, 374].

#### Regenerative therapies using genetic modification

As mentioned above, numerous genes are known to be involved in retinal dystrophy. Mutations in genes involved photo transduction or retinoid cycle (which are integrated) [375, 376], gene encoding proteins involved in ciliary function [377], and structural proteins have all been identified as causing the various forms of PR degeneration [378]. So, these disorders might have prone to be treated by gene therapy technology. A summary of the research into gene replacement therapy will be discussed in the following section.

##### Phototransduction and visual cycle pathway

**Fig. 5:**
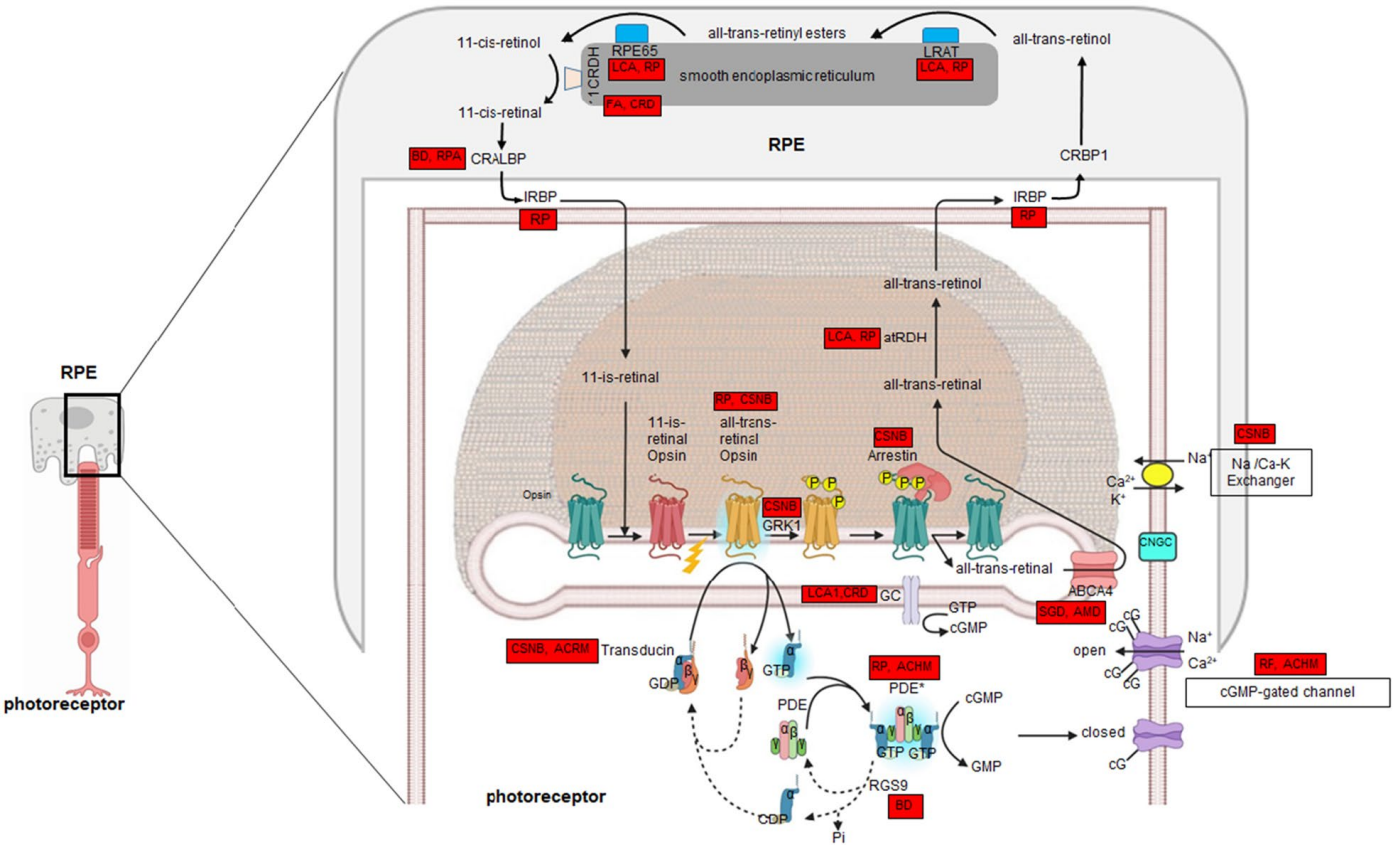
visual cycle pathway. Schematic representation of the phototransduction cascade and the visual cycle. In the left, the outer segment of PR was surrounded by the microvilli of the RPE apical membrane. In close, the biochemical events of RPE/ PR interaction have been presented. Upon light absorption by 11-cis-retinal opsin (inactive rhodopsin), the 11-cis- retinal rapidly is photo-isomerized to all-trans-retinal to form activated rhodopsin which in turn activates the heterotrimeric G-protein transducin and initiates the downstream signaling

The PR is liable for light retention and conversion to an electrical signal, next is transmitted to other neurons of the retina and thereupon to the brain [379]. The OS of PRs positioned bordering to the RPE layer is a specific fragment exclusively designed to accomplish phototransduction [380]. This mechanism involves a series of signaling proteins being sequentially triggered, leading to the eventual opening or closing of ion channels in the PRs membrane (Fig. 5) [381].

Phototransduction is the process by which, light is absorbed by a visual pigment molecule in PRs, and generates an electrical response. This cascade of cones and rods differs in their signal intensification and inactivation, which is based on their respective functions [382]. In this enzymatic cascade in the vertebrate and invertebrate, photons captured in the outer segments of the PR bring about the cascade, resulting in the closure of a fraction of the cGMP-gated channels in the cell membrane [383]. In other words, in the presence of light, the cGMP-gated channel flow Na^+^ and Ca^2+^ ions into the OS and thus play an essential role in phototransduction [384]. The overview of the molecular mechanisms of phototransduction indicated this cascade is initiated by light-induced isomerization of the 11-cis configuration of retinal (the visual pigment in opsin) to the light-insensitive all-trans form**.** Light absorption occurs by the chromophore as a particular isomer of vitamin A as visual pigments of rods and cones [383]. Light intake leads to phototransduction cascade, which results in PR hyperpolarization and arrest of glutamate secretion at their synaptic terminal [383, 385]. Entirely**,** activation of the phototransduction cascade can be broken down into four steps [386].

i. Activation of Rhodopsin: when a photon converts the ll-cis retinal chromophore of RHO to its all-trans isomer, photoexcitation is initiated consequently RHO molecule activation.
ii. Activation of the G-protein: The activated receptor protein (RHO) stimulates the G-protein transduction. In this process, guanosine 5´-triphosphate (GTP) is converted to guanosine diphosphate (GDP).
iii. Activation of the phosphodiesterase (PDE): In turn, activated transducing, activates the effector protein phosphodiesterase, which results in the activation of PDE and the hydrolysis of cGMP to 5'-GMP.
iv. Closure of Ion channels: The decrease in intracellular cGMP leads to the closure of the cGMP-gated channels and then rod cells become hyperpolarized.

Falling concentrations of cGMP also result in Ca^2+^ levels reduction in the outer segment consequently the exchange of Na/Ca-K continues to extrude Ca^2+^ from the outer segment.

The Gα-GTP activates phosphodiesterase (PDE), active PDE hydrolyzes cGMP (cG) to GMP. The decrease in cG concentration leads to the closure of the cG-gated channels, resulting in membrane hyperpolarization. New cG is resynthesized by guanylate cyclase (GC). Rhodopsin kinase (GRK) deactivates the rhodopsin by phosphorylation at multiple sites. Arrestin binds to the active phosphorylated rhodopsin to reduce its signaling to further transducin. The covalent bond between all-trans-retinal and opsin is hydrolyzed. Following this, all-trans-retinal is transported out of the lumen of photoreceptor disk membranes by the ATP-binding cassette transporter (ABCA4). Subsequently, the all-trans-retinal is reduced to all-trans-retinol in the reaction catalyzed by all-trans-retinol dehydrogenases (atRDH) and interphotoreceptor retinoid-binding protein (IRBP) and cellular retinol binding protein 1 (CRBP1) facilitate its transportation back to the RPE. In the RPE, all-trans-retinol is esterfied to all-trans-retinyl-ester by lecithin retinol acyltransferase (LRAT). RPE65 converts these retinyl esters to 11-cis-retinol. Subsequently, it oxidized to 11-cis-retinal by 11-cis-RDH. This molecule transports back to photoreceptors to complete the retinoid cycle by recombination with opsin to form the inactive rhodopsin. Diseases that result from mutations in proteins involved in the phototransduction cascade and the visual cycle are indicated in red boxes. Retinitis pigmentosa (RP), Congenital stationary night blindness (CSNB), Leber congenital amaurosis (LCA), Achromatopsia (ACHM), Bothnia dystrophy (BD),Cone-rod dystrophy (CRD), Stargardt’s disease (SGD), Age-related macular degeneration (AMD), Fundus albipunctatus (FA), Progressive retinal atrophy (PRA).

The genetically complex multifactorial cause of PR degeneration is exploring, attempting to uncover the cellular mechanisms underlying hereditary PR degeneration that would require knowledge regarding the enormous genetic heterogeneity of this disease group [38]. To date, the constructions and functions of visual phototransduction proteins and their roles in human retinal are also under explored. Many ocular diseases arise from abnormalities in retinoid related visual cycle proteins [387]. As mentioned above, signal transduction in the visual cycle happens due to a G protein-coupled receptor (GPCR) called opsin, which contains an 11-cis-retinal chromophore [388]. The combined changes in the receptor potentials of rods and cones trigger nerve impulses that our brain takes as vision [387]. G protein-coupled receptor; is light sensitive which contains an 11-cis-retinal chromophore [389]. One form of autosomal main RP (classified as rod-cone dystrophy) is attendant with a missense mutation, A346P, sited in the RHO gene. Data has been shown that this mutation restricts with normal regeneration of photoreceptors. Mutations consequential in a mutant RHO protein relates with the autosomal recessive disease. Thus, advanced central vision is a result of PRs cells shortage [390, 391]. RPE65 is a member of the carotenoid oxygenase family which is crucial for the proper function of the visual cycle. Thus, RPE65 mutation cause to prominent retinal abnormalities and dysfunction at birth. Moreover, RHO levels are significantly reduced in the RPE65 mutant [375, 392]. Mutations in RPE65 are associated with autosomal recessive retinitis pigmentosa. Over the past decade RPE65 model has been largely considered as an objective for gene therapy. Successful RPE65 gene therapy has also been displayed using the Swedish Briard dog model [393]. Recombinant AAV (rAAV) was injected subretinally at a variety of ages from 1 month to 4 years and resulted in significantly improved visual function (ERG), retinoid content, and visual behavior. Defects in the RPE65 gene lead to canine and mouse models of LCA which have recently been considered as models for gene therapy [394–396]. As to date, Voretigene neparvovec-rzyl (Luxturna, Spark Therapeutics, Philadelphia) was approved by U.S FDA for ocular gene therapy of RPE65 in 2017, which transduces some RPE cells with a cDNA encoding normal human RPE65 protein, making it possible to repair the visual cycle [397, 398]. Currently, human clinical trials using a similar vector are continuing. Clinical tails for ocular gene therapy were initiated through subretinal injection of an AAV2 vector carrying a normal human RPE65 cDNA for LCA2 patients (NCT00481546, NCT00516477, and NCT00643747) [399–405] which results in substantial visual enhancement [406, 407].

The common causation of STGD is a mutation in the ABC transporter A4 (ABCA4) gene. ABCA4 is located in the rim of the rod outer segment disc and the OS of the cones. ABCA4 deficiency leads to a toxic accumulation of all-trans-retinal leading to inherited retinal disease and eventually PRs death [408]. The major improvements in therapeutic applications for ABCA4 gene therapy have been established despite ABCA4 has been thought to be difficult to research [409, 410]. Allocca and colleagues injected AV2/5 containing Abca4 subretinally in an Abca4^−^/^−^ mouse model of STGD and observed improvement in the morphology and function of the retina for up to 5 months [411].

Interphotoreceptor retinoid-binding protein (IRBP) that is the major protein component of the interphotoreceptor matrix (IPM) and interacts with the cone “matrix sheath is secreted by PRs into the subretinal [412]. IRBP plays a role in the canonical visual cycle containing retinoid interchange between rods and RPE cells [413]. The reduction of IRBP in patients may contribute toward the advancement of diabetic retinopathy [414]. Because IPM acts as a physical barrier, so modifying IPM structure leads to a reduction of retina adherence to the RPE as a consequence of specific enzymes [316]. Subretinal injection of these enzymes (neuraminidase X and chondroitinase ABC) could lead to modify IPM and facilitating the lentiviral transduction of photoreceptors. This research group observed the increase in the number of GFP-positive photoreceptors when these cells were co-injected by lentivirus-Rho-GFP and neuraminidase. This finding showed that lentiviral vector transduction was improved in the presence of neuraminidase X [317].

##### Ciliary proteins (Intracellular trafficking proteins)

The photoreceptors are ciliated cells and have connecting cilium (CC), which is structurally similar to the primary cilium. Therefore, retinal degeneration due to PR death is caused by any defect or disruption in ciliary trafficking has huge consequences. Mutations in a variety of photoreceptor-specific and common cilia genes can cause rapid degeneration of photoreceptor OS and sensory cilia, consequently IRD [415]. Most of these genes are responsible for the OS development and thus involved in trafficking of specific OS-resident proteins in photoreceptors [416]. All the proteins associated with phototransduction are synthesised in the inner IS and then must be trafficked through the CC to reach the OS [417].

Precise regulation of all molecules delivered to the ciliary membrane is required for its correct development and function. Data have been reported that Bardet-Biedl syndrome (BBS) proteins seem to be involved in the trafficking of the vesicle from the golgi apparatus to the ciliary membrane [418] and in the actin dynamics to regulate ciliogenesis [419]. Viral AAV vectors transfected in the BBS1 mutant mice and subretinally were injected to restore BBS protein formation and RHO localisation thus showing trends toward improved electroretinogram function in mice [420].

There are many pieces of evidence that demonstrated that proteins encoded by the Usher genes play a crucial role in the cilia function of PRs [421]. Usher syndrome is a common form of syndromic retinal defect which is caused by mutations in the Usher genes. The most common form of Usher syndrome is Usher type 1B, which is caused by myosin VII*a* (MYO7A) mutation that is expressed in the RPE, and PR connecting cilium and synapse [422]. MYO7A is an actin-linked motor protein that plays a key role in the trafficking along filaments and clearance of opsin at the connecting cilium [423]. Thus, the mutation in MYO7A leads to the accumulation of opsin at the connecting cilium, consequently disk membrane morphogenesis impairment [424]. Data from a study showed that the defects in USH proteins are related to retinal degeneration diseases [425]. Thus, gene therapy is a valuable approach to rescue the Usher syndrome phenotype. MYO7A gene is 100 kb in size and making its AAV-mediated delivery problematic and leading to a focus on lentiviral delivery of MYO7A. For this, Hashimoto et al. applied lentiviral gene therapy to introduce MYO7A cDNA to the retina of null mice and observed appropriate levels of myosin protein and subsequently opsin clearance from the connecting cilium [426]. Dual AAV vector and fragment AAV (fAAV) delivery can transfer genes with a larger size than AAV alone. Dyka et al. observed that dual AAV/fAAV delivery of MYO7A to the retina of shaker-1 mice resulted in the appropriate gene expression [427]. Moreover, Lopes et al. observed that AAV2/5-mediated MYO7A delivery to the retinas of MYO7A -mutant mice corrected the mutant phenotypes in RPE and PRs. They could deliver full-length MYO7A to RPE and PRs [428]. Since PRs are the source of the initial expression of the disease, it is evident that this cell type needs to be adequately transduced in patients with Ush b1 [422]. So, it seems that the AVV vector is more suitable for the delivery of wild type MYO7A to both RPE and PRs. Besides, nanoparticle delivery of the Usher gene, thanks to their large packaging capacity, enhances the potential for Usher mutation treatment [429]. The results of animal studies finally have resulted in a phase I/II clinical trial which is currently in progress (NCT02065011), whears another clinical study has stopped not for safety reasons (NCT01505062) which was evaluating safety and tolerability of ascending doses of subretinal injections.

The retinitis pigmentosa GTPase regulator (RPGR) gene is located on the short arm of the X-chromosome. RPGR has a role in the trafficking of opsin from the IS to the OS of PRs [430]. Therefore, the mutation in the RPGR gene leads to mislocalization of opsin and then PR degeneration. The RPGR gene encodes a different form of RPGR. ORF15 isoform of RPGR is present in PRs [431]. RPGR interacting protein (RPGR-IP) is expressed in PRs at the connecting cilium and is required for localization of RPGR at this site [432]. Research has been demonstrated that mutations in the RPGR-IP are associated with LCA. As such, the first clinical trial was performed by Fischer and his colleagues in March 2017. They injected AAV2/8-mediated codon optimized human RPGRORF15 subretinally in males [433]. Zhao et al. showed that PR function could be improved using AAV-2-mediated expression of RPGR-IP, leading to the restoration of RPGR connecting cilium [434]. Rachel et al. showed that delivered myosin tail domain could retain ciliogenesis and PR function in rd16 mice [435]. Moreover, Beltran et al. applied the subretinal AAV2/5-mediated delivery of RPGR to the XLRAPA2 canine model and observed improvement of PR function [436]. Interestingly, clinical gene therapy for retinal neuroprotection in retinitis pigmentosa is in progress using AAV. (NCT03116113) was the first human study associated with X-linked retinitis pigmentosa (XLRP) which applied AAV8 vector-based gene therapy. Moreover, the safety and effectiveness of a recombinant AAV (rAAV2tYF-GRK1-RPGR) in patients with XLRP caused by mutations in ORF15 is evaluating in other clinical trial (NCT03316560). Furthremeore, a phase I/II clinical trial applying of AVV2/5 vector was initiated to carry PRGR in patients with XLRP (NCT03252847).

##### Structural proteins (disk morphogenesis)

Photosensory organelle OS is derived from a primary non-motile cilium. Disruption of the OS renewal process leads to a broad range of retina degenerative diseases. These OS contain a stack of closed flattened membranous sacs called disks. OS develops continuously through the OS renewal process, and the new disks displace present disks. This process required regulated membrane fusion which occurs to maintain an ordered arrangement of disks and physiological functions [437]. The Prph2 gene encodes a tetraspanin protein known as peripherin-2 (also known as peripherin/rds) that plays a critical role in the formation, maintenance, and renewal of these structres [438] and is located in the rim of the disk membrane [439, 440].

Peripherin/rds (P/rds), forms homo-oligomers or hetero-oligomeric structures with ROM1, which is another structurally related disc membrane protein. This protein is encoded by ROM1 gene and is necessary for disk morphogenesis [440, 441]. In mice, the absence of P/rds leads to RDS without OSs that eventually limited phototransduction capability and loss of PR viability [442]. Data have been demonstrated that the mutation in the P/rds gene results in retinal degeneration [443]. The subretinal delivery of AAV2 carrying the *Prh2* transgene could restore the correction formation of the photoreceptor disk [444]. Ali et al. have used an AAV-mediated delivery of PRPH2 *Prh*^*Rd2/Rd2*^ in the young animal and observed PR restoration functionally [444]. This group showed that OS induction was performed after AAV subretinal injection in the old animals [445]. Moreover, Georgiadis and colleagues showed that AAV-delivered microRNA to knock down Prph2 mRNA in the mouse retina [445].

In addition to the viral gene therapy, Cai et al. showed that nanoparticle-mediated gene delivery revives cone function to a near-normal level compared to naked plasmid DNA delivery. They found that DNA-nanoparticles can effectively transfect both mitotic and post-mitotic (terminally differentiated) retinal cells [446].

In conclusion, disruption of the proteins involved in the structure or function of PRs results in a wide variety of phenotypes, so that gene therapy may be used as a promising applicant for the improvement of these impairments. Gene therapy would be a useful application for the restoring of structures and functions in animal models.

In the current review, we tried to present all the significant reports regarding the status of gene therapy in IRDs. Some IRDs seem to be more feasible for future gene therapy, whereas further studies are necessary for other ones. Although the safety profile of gene therapy is the main limitation, adenovirus, AAV, and lentivirus have all been applied to replace disrupted genes in IRD [447, 448]. Overall, gene therapy has been investigated as a useful and potent therapeutic approach for the restoring of retinal function and treatment of visual impairments in clinical trials, with certain limitations (like safety aspects and clinical efficacy) that remain to be overcome.

#### Optogenetic approaches

Unfortunately, the death of PRs remains inevitable in most cases to date. The renewing of PRs or the restoration of retinal light responses provides the only retina-based strategy that could recover vision after PRs have been destroyed. The use of optogenetics and chemical-based photos witches has recently been one of the effective alternatives for applying these techniques [449]. Optogenetic therapy is a promising approach in a degenerated retina that has lost the light-sensitive photoreceptor cells, specifically in inherited retinal disease, incorporating neurobiology and genetic engineering techniques to restore vision through the supply of light-sensitive molecules to surviving retinal cell types that enable light-sensing through the residual neurons [2, 3].

Critical to the efficacy of this strategy is the availability of appropriate light sensors. These sensors have genetically expressed the gene of light-sensitive proteins, such as channelrhodopsin (ChR) or halorhodopsin (NpHR), to induce light-sensitivity in remaining retinal cells [3–5]. Since PRs are naturally sensitive to light, optogenetic tools can be used to restore retinal photosensitivity [6]. Cell membrane potential can be also modified by light consequencing. Cell’s intrinsic membrane photosensitive channels or receptors which are required in regulating the neuronal activity. These photosensitive molecules which are known as optopharmacological photoswitches contain two linked components including ligand and photoisomerizable group [7]. The revocable change of the photoisomerizable group from all-trans to cis configuration organized by light modifies the capability of the ligand to block/unblock channels or to activate/inactivate receptors [8].

The first optogenetic therapy to recover visual sensitivity by targeting retinal cell types was performed using an intravitreous injection of AAVs expressing ChR2 with a specific promoter sequence [2]. Optogenetic approaches commonly target remaining cells that represent at least a minor portion of IRD. Garita-Hernandez and colleagues showed that optogenetically-transformed photoreceptors could restore visual function in blind mice lacking the photoreceptor layer, as well as behaviourally [450]. Moreover, visual function was restored via co-expression of ChR2/ HaloR in RGCs in the retina after the death of rod and cone photoreceptors [451]. Also, Busskamp et al. showed the visual restoration in the retina of animal models of RP using the chloride pump halorhodopsin, [9]. To date, trials suggest that optogenetic therapies show great potential promise in patients with advanced hereditary retinal disease to restore vision, and further clinical research is required as the next major step in advancing the field. Capabilities of visual function can be enhanced using of optogenetic therapy in combination with other therapies such as cell therapy and neuroprotection in preclinical animal studies [2, 4, 10, 11].

### Cell based therapies

As mentioned before, following the progression of visual impairments, the neural retinal layer is gradually destroyed and therefore, the administration of neuroprotection drugs or gene therapy alone could not improve the visual abilities, and the injured cells need to be replaced by healthy ones.

Cell therapies are a popular treatment strategy for the late stages of PR degeneration, where the conventional protein or chemicalbased methods are not effective. When an incompletely differentiated cell type is transplanted to a certain area of the body, signals received from the microenvironment can be expected to affect cellular phenotype. This variability in potential cell fate makes some challenges in applying stem cell therapy, which will be discussed later. Cell therapy can improve transplant outcomes via two main approaches. First, the injected cells could replace damaged cells and replicate their normal activities in the recipient site. Second, there is a possibility of secreting biological factors that provide a modified microenvironment supporting tissue regeneration, renewal of impaired cell activities, and host neuron survival, as discussed previously. In the following sections, different cell sources for differentiation and replacement therapies will be highlighted.

#### Current differentiation strategies

Cell therapy is one of the promising treatments suggested for replacement in some advanced cell impairments. Different cell sources have been used in transplantation according to the accessibility of the cells source, ethical issues and the number of cells required for transplantation. Also, the transplantation of the cells in an appropriate stage of development is another area of consideration.

A differentiation process can occur through three different mechanisms as follows: spontaneous, direct, or co-culture methods. In spontaneous differentiation, the cells were undergoing differentiation based on their own growth factor secretion [452, 453]. Therefore, a variety of cells were produced, and the efficiency of differentiation was decreased due to the variation of the produced cells. The direct differentiation process was taken through stepwise modulation of signalling pathways using specific activators or inhibitors. In most experiments, Wnt and BMP pathway inhibitors were used to direct the eye field differentiation [454–456]. Therefore, the cells were directed toward anterior neural ectoderm, which is continued by eye field differentiation. Activation of tyrosine kinase pathway by bFGF, EGF, and IGF could improve the yield of retinal differentiation [457]. Also, the reports have shown that according to in vivo developmental events, the co-culture system could mediate cell differentiation [458, 459].

#### Different cell sources

##### Fetal cells

Cells obtained from the fetus are capable of developing into the various cells of a defined tissue. Fetal neural stem cells (fNSCs), also known as neural progenitor cells (NPCs), can be isolated from fresh discarded human fetal brain, or eye, in which case they are known as retinal progenitor cells (RPCs). These various neural progenitor cell types have already undergone considerable fate specification relative to pluripotent stem cells and, therefore, may or may not need to be further directed towards specific cell fate via various differentiation protocols (Table 2). Administration of TGF-β3 [460] or Retinoic acid/T3 [461] appears to improve in vitro and in vivo differentiation of the fNSCs into photoreceptors. Also, the co-culture of human RPCs with mouse retinas for two weeks influenced the differentiation efficiency such that there was two-fold increased expression in specific markers of retinal cells [462]. hRPCs cultured in the presence of neuro-supplements N2 and B27 could maintain their innate state [463], at least transiently. Das et al., transplanted human fetal neuroretinal cells for the patients with advanced RP. A 40-month post surgery evaluation indicated no detrimental effects in clinical outcomes [464]. In the other report, transplantation of fetal retina with RPE survived for one year showing continued improvement in visual acuity without rejection response [465]. The first FDA approved clinical trial using fetal RPCs (fRPCs) was carried out by Klassen et al. for RP patients (NCT02320812) with accetable outcomes which is followed to phase IIb study (NCT03073733). Similar efforts were also carried out and 6–24 month follow-up showed biosafety and feasibility of hRPC transplantation which lead to progress towards future clinical trials [466]. Nevertheless, a range of considerations, including ethical and regulatory limitations imposed on the sourcing of cells from discarded fetuses, as well as the limited yields of cells of this type, have convinced many investigators to utilize adult stem cells whenever possible or, alternatively, pluripotent stem cells.

**Table 2:** Differentiation methods of human stem cells toward photoreceptors

Cell source	Differentiation method	Culture medium condition	Cell types derived in vitro, specific markers (days)	Preclinical or clinical studies towards cell therapy
Human fetal neural stem cells (fNSC)	Direct differentiation	TGF-β3	Photoreceptors, OPSIN (D15)	The transplanted cells in the rat model showed the extensive migration into the lesion area of the retina [460]
Human exfoliated deciduous teeth (SHEDs)	Direct differentiation	N2,B27, FGF2, noggin, Dkk1, IGF-1, Shh, T3, RA	Photoreceptors, RECOVERIN, CRX, NRL (D24)	Injected SHEDs into the mouse eyes could differentiate into rod- and cone-like cells; retinal function improved, and photoreceptors were rescued for 3–5 months [468]
hBM-MSC	Spontaneous differentiation, Direct differentiation	FGF2, EGF, BDNF	Photoreceptors, RHODOPSIN (D7)	Phase I trial was done in 3 patients with RP and two patients with cone-rod dystrophy 10 months after transplantation revealed no detectable structural or functional toxicity, demonstrating the short-term safety of the transplantation (NCT01068561) [492]. Phase II study enrolled 20 RD patients. Findings showed vision-related life quality improvement 3 months post treatment (NCT01560715) [493]
hRSCs	Direct differentiation	FGF2, EGF	Rod photoreceptors, RHODOPSIN (D21)	After transplantation into the mouse eye, the cells showed photoreceptor morphology and expressed RHO marker; also able to integrate into different neuronal layers at appropriate developmental times [470]
hRPCs	Direct differentiation	N2, B27, FGF2, EGF	Photoreceptors, RHODOPSIN and RECOVERIN (D14)	Transplantation in RCS rats revealed the integration and expression of specific markers. Also clinical study in retinitis pigmentosa 8 patients after 1–2 years post-transplantation clarified no obvious autofluorescence destruction in the macular area and also significant improvement in visual acuity. Although poor visual function of the patients prior to the study was detected. No immunological rejection was not observed after transplantation in both animal study and clinical trial [466, 494, 495]
hESCs	Direct differentiation	N2, B27, FGF2, IGF-1, Dkk1, Noggin, COCO	Con Photoreceptors, CRX, S-OPSIN (D21)	Injected cell clamps into the vitreous of neonatal (P1) mice were presented in the PR nuclear layer by expression of S-opsin and the same morphology of host PRs [496]
hiPSCs	Direct differentiation	N2, B27, IGF-1, Dkk1, Noggin, DAPT, FGF1 and 2	Rod Photoreceptors, RECOVERIN (D90) and RHODOPSIN (D120)	Two weeks following transplantation into neonatal retinal degenerative Crb1 mutant mice, PR precursor cells were integrated into the outer nuclear layer and differentiated into morphologically and immunohistochemically recognizable PRs [497]
hESCs/hiPSCs	Direct differentiation	N2, B27, Noggin, BDNF, CTNF, Insulin, DAPT, RA	Photoreceptors, CRX, NRL, NR2E3 (D90-100)	Three weeks post transplantation in the adult rd1 eye, the expression of mature PR markers and functional analysis indicated visual improvement [489]
hESCs/ hiPSCs	Direct differentiation	N2, B27, FGF2, IGF-1, Dkk1, Noggin	Photoreceptors, CRX, NRL (120) RECOVERIN, RHODOPSIN, S-OPSIN (60)	After transplantation to the sub-retinal space, the cells begin to move into the retina and expresses mature PR markers [454, 455]
hESCs/ RPE	Co-culture differentiation	N2, B27, FGF2, IGF-1, Dkk1, Noggin, Shh	Photoreceptors, RHODOPSIN, S-OPSIN (D40-50)	Following transplantation of Shh-treated retinal cells in eighteen adult albino rabbits for 4 weeks showed ERG improvement, and also cell incorporation into the retina, and finally visual restoration [491]

##### Adult cells

Adult stem cells are originated from the adult body and able to renew or repair generally all cells of the specific tissue with the same origin. The most used cells in the experimental studies include various populations of MSCs such as BM-MSC, umbilical cord blood-derived cells (UCBCs), dental stem cells (DSCs) and also the stem cells derived from another part of the eye like cilliary body epithelial cells (CEs), iris pigment epithelium cells (IPECs), and RSCs. There are some reports illustrating the differentiation ability of MSCs toward photoreceptors (Table 2). Genetically modification of Wharton’s jelly of umbilical cord MSCs with erythropoietin (EPO) gene in the presence of an induction medium containing taurine could increase rod photoreceptor differentiation yield [467]. Transdifferentiated cells from human exfoliated deciduous teeth (SHEDs) into photoreceptor-like cells according to a factor-cocktail protocol expressed specific markers of recoverin and opsin, and some in vitro Ca^2+^ activity and also maintained their survival after transplantation in rat [468]. The induction potential of the cells in co-culture systems also might conduct stem cell differentiation. The presence of hRPE in a co-culture system conducted BM-MSCs differentiation towards photoreceptor-like neurons expressing PKC and opsin [469]. Retinal stem cells derived from CE are another cell source for cell therapy experiments. The evidence showed that CEs isolated from the retinal ciliary margin's pars plicata and pars plana could maintain their multipotency potential by EGF and FGF. The observations showed that during 28 days, transplanted cells into the mouse eye were located in the photoreceptor layers and expressed specific markers of PRs [470]. Also, the CEs enriched by size, granularity, and low expression of P-cadherin could differentiate to rod PRs in the presence of exogenous supplements such as taurine and retinoic acid [471]. MSCs have been broadly investigated in clinical application for retinal disorders. In a non-randomized phase I clinical trial of 14 patients with RP intravitreally transplantation of BM-MSCs showed an improvement in visual functions [472]. In Phase III clinical trial, the umbilical cord-MSCs (UC-MSCs) were introduced for RP patients. At 6-month follow-up, about half of the patients showed improvmenet in visual criteria. In parallel, intravenous administration of UC-MSCs indicated acceptable results with the successful efficiency of around 90% [473, 474]. The similar results have been also achieved by BM-MSCs [475–477]. Sub-tenon injection of placenta-derived mesenchymal stem cells (PD-MSC) rescued the RGCs and improved the visual acuity [478]. The MSC derived from wharton’s jelly (WJ-MSC) significantly improved best-corrected visual acuity (BCVA), visual field, and ONL thickness in phase III clinical trial [479]. The visual performance improvement without deterious side effects was also reported via subscleral space injection of Adipose-derived stem cells (ADSCs) [480].

##### Pluripotent stem cells

Pluripotent stem cells (PSCs) originated from the human blastocyst stage of embryo development (hESCs) or reprogrammed mature cells (iPSCs) are considered as an unlimited cell source for the treatment of degenerative diseases. Evidence began to accumulate that hESCs differentiation protocols towards the neural retinal cells, including PRs (Table 2). As described earlier, according to direct differentiation methods, inhibiting the Wnt and BMP pathways would improve anterior neural differentiation [454, 481]. In this method, the ESCs were cultured as floating aggregates under serum-free conditions. The addition of DKK/Lefty to the culture medium could increase the efficiency of anterior neural differentiation. In parallel, inhibition of Wnt/BMP pathways provides a permissive environment for forebrain development. Consistent with this, other studies were followed by some modifications to improve the differentiation efficiency. For example, enrichment of the culture medium by neuroprotective factors N2 and B27 and some growth factors like bFGF and EGF could improve the maintenance of retinal differentiation [452, 455, 482, 483]. Although, some others components, such as taurine, Sonic hedgehog (Shh), or retinoic acid, could affect the differentiation potential of hESCs/hiPSCs towards neural retinal cells, particularly photoreceptors [484–488]. Cells exit from the cell cycle by notch inhibitor DAPT, also increases the PR differentiation [489, 490]. As explained earlier, the co-culture system directs the ESCs based on secreted factors distributed by the induction of adjacent cells after a certain period of time. The co-culture of hESC-derived RPCs with rabbit RPE could modulate rod photoreceptor differentiation by Nrl expression [491]. The recoverin + cells derived from hESC- RPCs have appeared in retinal explants of adult mice on 0.4-μm filters during the co-culture system [454]. Some previous reports demonstrated the stromal-derived inducing activity (SDIA), which could be used in co-culture systems. The hESCs/hiPSCs were also induced to the eye field specification co-cultured with stem cells from apical papilla (SCAP) [458]. There is some evidence that high-density culture of hESCs/ hiPSCs leads to the secretion of Wnt and BMP signalling inhibitors such as DKK and Noggin, which is followed by eye field specification [452, 453].

##### Retinal organoids

An organoid is a 3D cell mass containing stem cells in which cells are self-organized similarly to in vivo counterparts [498, 499]. Organoids can be derived from different cell sources, including stem cells, embryonic/pluripotent stem cells, or even a single adult stem cell [500]. They can be utilized for understanding the characteristics of stem cells, the development of tissues during embryogenesis, the regeneration processes in the adult tissues, providing a platform for drug screening or transplantation to damaged tissues [501]. Retinal organoids currently represent the great in vitro quality characteristics to generate high amounts of PRs that can be further developed towards cell-based therapy applications [502].

The ability of hPSCs to generate retinal organoids open a road toward the generation of clinical cell populations for replacement therapies in the eye [483, 487, 503, 504]. Pioneer studies reported by Sasai research team showed the generation of 3D retinal organoids that closely follow in vivo retinogenesis [487, 505]. To improve the efficiency of retinal organoid formation, a trisection protocol was suggested that increased the yield of retinal organoids to 183% [490]. Also, the combination of 2D/ 3D differentiation protocols is useful to increase the efficiency of differentiation. Studies in these protocols continue the isolated optic regions from the adherent cultures to suspension for further culture [506, 507]. Some results showed that the direct co-culture of hESCs with different mesenchymal cells could improve the efficiency of optic vesicle and optic cup formation [508].

The presence of all major retinal cell types, including PRs, in a highly organized construction, had been reported in pioneer retinal organoid publications [487, 509]. In addition, the formation of highly specific structures like the ribbon synapse [506, 510] and OS [511, 512] was also described. Data have been reported that retinal organoids, including PRs showed the reactivity potential to light stimuli, and therefore, they pass the information to the inner retina such a physiological manner [513, 514]. 3D retinal organoids offer a potential tool to either sort out the PR precursors [515] or directly use them like a sheet of retinal organoids for transplantation [516].

#### Cell delivery approaches

Regardless of the cell type, the methodology of the donor cell delivery is an important topic that should be considered. From the surgical point of view, there is a high chance of transplantation success in the retina as the eye is considered to be an immune privilege organ and its accessibility [69]. To achieve a successful cell replacement, not only the isolation and generation of the desired cells but also the functionality of the survived cells after delivery to the target location is required [517]. The main strategies for cell delivery therapies are categorized into retinal cell suspension and tissue (sheet) transplantation.

##### Retinal cell suspension

Since cell suspension transplantation is a controllable manner for experimental and preclinical studies, until now, almost all of the strategies used for cell transplantation have been focused on cell suspension transplantation. Other than the selected route for cell delivery, which has been discussed before in this review, in most studies, the cell suspension was injected in the eye space by a syringe with common gauges 30–31. The Hamilton syringe is the most commonly used device for this purpose. The rate of cell delivery and the probably shear stress caused by injection on cell survival and cell deformity are important problems that should be considered [518–520]. Cromel’s study showed that the hRPCs carried by a gelatine-based gel through the 31-gauge needle revealed high levels of the survival, proliferation, and expression of the specific factors compared to the cells that were injected in PBS as the carrier [521]. Evidence showed limited successful transplantation in suspension manner because of the low number of integrated cells or cytoplasmic exchange. [145, 522, 523]. Therefore, the transplantation of the retinal sheet could be an effective replacement strategy.

##### Tissue (sheet) transplantation

Conserving the donor PRs layered organization and structural channel guidance, retinal sheet transplantation has been studied as an alternative strategy for cell delivery in PR degeneration diseases [524]. The grafts derived from retinal-sheet transplantation, which are correctly located at the sub-retinal space, are expected to develop outer and inner segments facing the host retina [525]. Additionally, it is observed that the graft survival of the sheet transplantation will enhance significantly in comparison with the cell suspension method (6–10 months post-transplantation [526] to 3–4 months post transplantation [72]).

However, treatment outcome mostly depends on the degeneration stage. In the early stage, single-cell suspension might support the remaining survived PRs, while at the late stages, a structured PR sheet will improve synapse formation between the donor and host retinal cells.

Maintaining the architecture of the transplanted cells, tissue-engineered scaffolds consider an efficient modality for the donor cell delivery [527]. These scaffolds can conduct cell behaviour such as proliferation, adhesion, post-transplantation migration, ECM synthesis and differentiation while delivering the bioactive materials [528]. In order to design a suitable scaffold for cell delivery purposes, some key factors to consider are biocompatibility (retain donor and host cell viability, not produce immunogenic response) [529, 530], biodegradability (degrade after ECM production by donor cells) [531, 532], mechanical properties (flexible with low young modulus to mimic the retina tissue and mechanically strong to withstand the surgical implantation) [533–536]. In addition to the mentioned factors, scaffold architecture is also key to achieve successful cell-scaffold delivery. To illustrate, appropriate scaffold porous structure and (surface topology can allow diffusion of nutrient and conduct the graft formation [537–541]. To date, solvent casting, polymerization and grafting, phase separation, freeze drying, micro fabrication, and electrospinning are the main manufacturing methods used for these scaffold fabrication [542]. Natural and synthetic materials can be used as scaffolds substrate [543]. Some of the most common biomaterials applied in scaffolds are: natural polymers extracted from humans or animals (Hyaluronic acid, collagen, gelatine, fibrin) [544–547], natural polymers extracted from bacteria or plant (Hyaluronic acid, alginate, cellulose) [548–550], synthetic polymers (poly-L-lactic acid (PLLA), poly lactic-co-glycolic acid (PLGA), poly glycerol sebacate (PGS), poly e-caprolactone (PCL), poly ethylene glycol (PEG); poly methyl methacrylate (PMMA) in polymer composites [551] or alone) [552] and decellularized tissue (Bruch’s membrane, inner limiting membrane, amniotic membrane) [553, 554]. Therefore, an appropriate selection of materials is a fundamental consideration in developing supportive scaffolds. As such, different functional groups on the surface of biomaterials can simulate different biochemical signalling pathways. For example, Behtaj et al. reported that a combination of advantageous surface and bulk properties of the aligned electrospun PGS/PCL scaffolds could promote RPC attachment and growth [555]. The readers are directed to the comprehensive reviews [556, 557] on the subject of scaffolds transplantation design aspects.

During scaffold based cell delivery, protection of host tissue and minimization of surgical invasion upon transplantation as well as exploiting a cost-effective fabrication should be considered. For example, the invention of an injectable scrollable scaffold, which maintain the scaffolds unfold prior implementation could improve the transplantation efficieny. Redenti et al. have designed a micro-fabricated PGS scaffold, which demonstrated long-term mRPC survival and exhibited mature marker expression in host retina. The PGS–mRPC composites retained sufficient elasticity to be scrolled and then injected via the sclerotomy into the subretinal space [558]. An alternative strategy to modulate the interaction between the donor cell and the scaffold surface is surface modification such as laminin coating [559], plasma technique,s and wet chemicals [560]. Oxygen and air plasma were observed to generate a more hydrophilic surface where attachment and growth of a human RPE cell line were enhanced [561, 562]. Laminin coated scaffolds were also displayed minimum folded/rosette shape and facilitated subsequent cell adhesion [559, 563]. Likewise, the biodegradability of the implanted scaffolds is desired due to the unique microenvironment of the sub-retinal space and the possible damage to the host retina [564, 565]. Gandhi et. al have demonstrated the complete degradation of the fibrin-gel scaffold placed in the subretinal space within 8 weeks, with no damage to the neurosensory retina or endogenous RPE [566]. After degradation the sub-retinal space, which was generated artificially during transplantation, would disappear thus resulting in retina reattachment.

As mentioned earlier, PR cells have a highly apical-basal polarized orientation. One of the challenges of PR transplantation is the polarization of the donor PR cells after grafting into the sub-retinal space. This might not happen via bolus injection of the cells. Jung et al. [567] have developed a “wine glass” scaffold design (an array of cup-shaped PR capture wells that funnel into a microchannel) made from non-biodegradable polydimethylsiloxane and biodegradable PGS. This design was able to capture human pluripotent stem-cell-derived PR cell bodies and guide the basal axon extensions. Interestingly, several scaffolds for cell transplantation addressing the PR regeneration are in clinical trials assessment.

#### Challenges for cell-based therapies

##### Production of a complex retinal tissue

Previous studies have shown the injection of cell suspension into the subretinal or interavitreal space which has been reviewed by Gasparini et al. [69]. Nevertheless, the injected cells showed low survival and limited ability to reorganize into a functional monolayer and often failed to regain a fully differentiated phenotype [564, 568]. Therefore, transplantation of retinal cell layers could be replaced to improve transplantation efficiency. Also, different types of cells were impaired in some advanced visual disorders, and effective improvement was provided by transplantation of the retinal tissue structures.

Few studies have been published on the transplantation of retinal tissue containing RPE and PRs. The first one, reported by Armanat et al., assessed the transplantation of human retinal and RPE sheets in nude rats. Twelve months later, the RPE maintained its normal sheet morphology and supported the transplanted retinal laminations in some cases, but most PRs were developed as rosette-like structures [569]. These transplanted retinal sheets also presented some functional recovery in 30% of the rd1 end-stage retinal-degeneration mice with a higher percentage of recoverin expression [570]. Besides the specific marker expression, the connections between different neural cells are very significant after cell transplantation. Engrafted PRs from transplantation of fetal retinal sheets could be arranged as the rosette-like structures [521] and connect with the host retina [571]. Some years later because of some immune rejection aspects, most of the studies have been adjusted on 3D neural retinal sheets derived from pluripotent stem cells. They survived in the host retinal and expressed specific markers of different neural retinal layers. Additionally, direct synaptic connections were also detected [572–574]. Although the hPSC- retinal organoid consisted of PR precursor cells, after transplantation, rod and cone cells were derived from transplanted organoids, and the rosette-like structures were detected within some inner cell layers. Also, the organization of differentiated retinal cells were not followed the correct natural structures [516]. A one-year follow up showed the high competency to mature and respond to light stimulus [575]. Also, the recent result published by Salas et al.showed that the cell therapy by co-transplantation of RPE and RPC conserved the survival and function of ONL superior to RPE or RPC [576]. Therefore, as the results presented, transplantation of complex retinal tissues could help to improve the results of future clinical applications and need to evaluate in more detail.

##### Optimization of transplantation procedure

*Folding or damaged structures* Besides increasing studies about the complex retinal sheets formation and characterization, the delicate architecture of the retina and the orientation of the grafted structure requires further extensive investigations. A few recent studies have released their results about the possibility of the 3D structure transplantation at the correct orientation. According to their findings, the folded or damaged sheets were excluded from the experiments, and just the best position sheets were recommended for long-term follow-up [577]. Although this report is limited to the RPE layer injection, there is no evidence of the optimization of retinal sheet transplantation in the posterior part of the eye. In order to achieve highly retina’s architecture, control of the retinal sheet transplantation site is an important issue that should be noticed.

*Transplantation techniques and instruments* Some difficulties, including procedure visualization through small pupil windows and relatively larger lenses, are associated with sheet transplantation into smaller eye animal models, such as mice and rats. The larger vitreous cavity ratio in larger eyed animals, such as monkeys and rabbits, provides more space to insert devices while increases the experimental costs [578]. In both cases, successful transplantation can be achieved when the delivery instrument would preserve the delicate tissue during transplantation manipulation, and handling of the tissue into the position can be easily performed once they are in the subretinal space[579]. Several customs made devices have been developed to facilitate the surgery. The following paragraphs explain some of these delivery instrument designs.

Kamao et al. have developed a surgical device for subretinal transplantation of hiPSC–RPE cell sheets into 12 rabbit eyes. This design consisted of a custom-design hand piece and a cannula. The cannula itself has two parts: a medical 20-gauge intravenous catheter, which can be reinforced using a custom-design blunt needle and a medical 1 mL syringe inserted into the plunger. Implementing the surgical device, the surgeon would be capable of loading and ejecting the grafts with a single hand [577]. An alternative explant injector was built for the controlled delivery of the delicate sheet into the subretinal or intraretinal space through a vitrectomy approach. The significant novelty of the proposed device is the invention of a perforated carrier platform at the tip of a cannula, which is protected via a second cannula and easily introduced through sclerotomy. The driven pressure is provided with a syringe attached to the plastic tubing. The same group has also proposed a modified instrument with a smaller size and an angled tip for human subretinal surgery [579]. Some designs have utilized MEMS manipulators for cell sheet transplantation [580]. MEMS manipulators basically consist of three parts: a head, arm and base with interconnection. Employing the same principles, an improved device has been constructed such that both MEMS manipulator and the needle can be controlled for precise positioning of the RPE sheet into the subretinal space. The movable MEMS manipulator and needle parts were driven by a driving bar to move MEMS manipulator itself and expose the head of MEMS manipulator, receptively [581]. Several efforts have been made to design an instrument capable of preserving RPE cell monolayer polarization required to restore vision. A study has developed a vitronectin-coated and mesh-supported submicron parylene-C membrane to create a hESC-RPE confluent monolayer for subretinal implementation. The proposed device was found to preserve transplanted cells as an intact monolayer for 1 month in the subretinal space of the minipig eye [582]. Another study has designed and fabricated a bullet shaped cell carrier system made from rigid-elastic polyester membrane with 0.4 μm pores [583]. They have found that gelatine encapsulation of the implants providing more control over the implementation process compared with “naked” implants. Moreover, a custom-designed shooter consisted of an angled nozzle, a non-stick plunger and an actuator was built to position the implants in the subretinal space. The newly developed nozzle is flattened with drills on the surface. The proposed surgical techniques granted safe transvitreal delivery of the implant into the subretinal space of rabbits. Readers are encouraged to refer to references for more details about each design.

##### Appropriate cell number and cell stage during transplantation

Although there is a growing evidence showing the optimization of experimental protocols to achieve RPCs, insufficient donor cells for cell therapy studies due to the limited proliferative capacity of these cells is a critical point that needs to be resolved for RPC [584, 585]. The optimal optogenic stage of PRs could affect the rate of integration following transplantation. A growing body of studies demonstrated that the RPCs expressing postmitotic marker Nrl had higher integration potential into host PR cells when they were compared to the neural retinal cells stated at earlier or later stage of development [522, 586, 587]. After transplantation, the rod precursor cells exhibited morphological characteristics such as outer segment structure and expressed some synaptic proteins like bassoon and ribeye. By retaining the critical properties, the cells were integrated into the ONL of the host retina, and the visual acuity of the animal model was improved [574, 588]. The evaluation of the synaptogenesis following transplantation is important, and recently a new method named QUANTOS has been reported evaluating synaptogenesis during the development and regeneration of the retina. This method provides the quantitative and qualitative evaluation of synapses after cell transplantation [589].

Another challenge with cell transplantation, which has been addressed by recent protocols, is that transplantation of a heterogeneous population leads to greatly reduced numbers of integrated cells. Recently, some approaches were assessed to address this limitation, including the enrichment of suitable cells for integration in the host retina. The results clarified that the cellular homogeneity, such as enrichment of the rod photoreceptor via specific cell surface marker CD73 could affect the transplantation outcome [590, 591]. A cone biomarker panel (SSEA1 − CD26 + CD133 + CD147 +) is also introduced for the purification of L/M-opsin cones. In contrast, there is no preliminary in vivo evidence about their behaviour after cell transplantation [463]. A two-step immunopanning of antimacrophage antibody, followed by anti-Thy1 antigen, facilitated retinal ganglion cells' enrichment for further analysis [592]. Based on cell therapy's important role in replacing the damaged or lost cells, identification and introduction of specific cell surface markers should be magnified.

##### Establishment of Good Manufacturing Practices (GMP)-compliant protocols for cell preparation

Although a large number of studies have been proceeded to improve the achievement of in vitro RPCs or PRs especially derived from hESCs/ hiPSCs, the important issue that should be considered in experimental and preclinical studies is the differentiation of the cells according to GMP protocols which there is some few evidence. As a pioneer study, Wiley reported the generation of hiPSCs from a patient with inherited retinal degeneration under ISO class 5 cGMP conditions. The neural epithelium was derived from hiPSCs by replacement of animal-derived matrigel and FBS with human ECM mixture and cGMP human grade serum, respectively. Further molecular analysis also confirmed the innate characteristics of cGMP grade hiPSCs- photoreceptor precursor cells [593]. Also, transplantation of the cGMP-manufactured human iPSC lines derived PR showed successfully integrated into the subretinal space of the immunodeficient host mouse eyes [594]. As the protocols develop, the field needs to keep in mind is that propagation and maintenance of the cells under GMP grade conditions is a critical issue that should be considered.

##### Type of donor cell

The cell sourcing is one of the key factors affected the final results of cell transplantation. In this regard, HLA matching which means the similarities of the leucocyte antigen between recipient and donor cells has a significant as well as population characteristics should be addressed. Some evidance showed that in compare to autograft BM-MSC, the allograft or xenograft transplantation could change the retinal functionality and anatomical structures in animal model. Beside the morphological changes, microglial activation and migration to surround donor cells in allotransplantation and xenotransplantations, which was followed by CD45^+^ cells recruitment, the expression level of anti-inflammatory proteins was significantly lower in autograft group [595]. In addition, some individual characteristic of donor such as weight [596], age [597] and the rate of health [598] might affect the transplantation efficiency. Though there is no report on retinal outcomes.

### Visual prosthesis

At the late stages, when the approaches mentioned above are not effective anymore, the visual prosthesis can be used to create electrical impulses to substitute the electrical stimulation that would normally be created by the PRs. Transcorneal electrostimulation would help patients who still have functional PR. Thus, bypassing failing parts of the visual pathway, the image information would be delivered to the healthy parts of the natural visual system [245, 248, 599–601]. The idea of the visual prosthesis is based on the production of phosphenes (white or coloured sparks of light with a structured appearance). A reliable phosphene can be obtained by careful optimization of electrical stimulation parameters [602]. There are two approaches to stimulate retinal ganglion cells: (1) Directly (general prosthesis) or (2) Indirectly (optical-sensor prosthesis) via the surviving network of retinal neurons.

The general prosthesis is composed of two modules: external and implanted. The external module of a general prosthesis consists of a video camera, usually worn on eyeglasses, which is responsible for video capturing, image pre-processing and hence key image data transmitting to the implant via a wireless interface. Photodiode arrays [603], charge-coupled devices (CCDs) [604], and complementary metal-oxide semiconductor (CMOS) have been considered as image and video cameras for external modules of the general prosthesis [605]. Data first will be captured from the outside world, pre-processed and then reduced to the amount compatible with the processing capabilities of the implanted device. In order to minimize the risk of infection, data and power convey mostly will be carried out via a wireless telemetry implemented by capacitive links. An embedded controller commands a microelectrode array to convert transmitted data into electrical activity with customized amplitude and pulse widths. Engineers, in collaboration with specialists in clinical and surgical fields, are working together to develop the visual prosthesis that can allow greater replication of the natural neural stimulation of the retina. Some of these efforts have been dedicated to alternative microelectrode materials that would ameliorate electrical signal production. Conductive polymers such as polypyrrole (PPy) [606], poly 3, 4-ethylenedioxythiophene (PEDOT) [607–610], and polyaniline (PANI) [611] in the form of a conductive coating or electrode material have been reported to enhance electrode functionality by lower impedance and higher charge injection limit. Nanomaterial such as carbon nanotubes (CNTs) [612–614], nanocrystalline diamonds (NCDs) [615–617] and silicon nanowires (SiNWs) [618, 619] have also offered enhanced properties to be presented as a promising substitute for electrode materials. Replacing metal electrodes with nanomaterials will improve electrochemical properties, neuron-electrode interaction, and electrical activation of retinal neurons due to unique surface morphology and charge injection mechanism [601]. General prosthesis under trade name Argus II epiretinal device (Second Sight Medical Products, Inc., Sylmar, CA, USA) is commercially available as the first camera-based visual prosthesis [620].

Principally, the resolution depends on the number of stimulation sites. However, from the engineering point of view, there is a great interest to maximally benefit from the natural visual system [604]. In this regard, various stimulation sites are considered: direct stimulation of visual cortex via cortical implant [621], stimulation of optic nerve via cuffs that encircle the optic nerve [622], stimulation of the lateral geniculate nucleus [623] and stimulation of the retina [624]. To interface the retina and the electrode array, the implantation would occur at three locations in the visual pathway: the epiretinal [620], the subretinal [625], and the suprachoroidal spaces [626, 627]. Although each method has its own advantages and disadvantages, the appropriate approach will be determined depending on the cause of vision loss and the patient’s particular vision perception.

To indirectly stimulate the RGC, the rest of the system besides PRs (cornea, iris, and lens) must be healthy and functioning. The basic architecture of a visual prosthesis, which is compared to the function of PRs, comprises a micro-photodiode array capable of converting incident light into electrical activity and delivering the resulting electrical stimuli to the retinal ganglion cells. This prosthesis is, thus, much more dependent on the qualities of the natural visual system [603, 628]. Fixation of the implant occurs at the sub-retinal space where the bipolar cells (the next surviving layers of neurons in the visual pathway) present in the closest proximity [629, 630]. Contrary to the general prosthesis, an optical sensor prosthesis is, indeed, a single component with no need for an external camera. Thus, fewer components need to be implanted, and natural eye movement would be used to locate the object [631]. However, in the absence of an external video camera, this system requires relatively high levels of light to effectively stimulate the adjacent neurons [628]. Two clinical trials are completed on optical sensor prosthesis under trade name Alpha IMS [620, 625, 632, 633] and ASR microchip [630], in which Alpha IMS is commercially available.

Implantation of the photodiode array component at the sub-retinal space has some drawbacks, such as separating pigment epithelium and hurdle of blood flow from the retina. To address these limitations, Benfenati et al. has recently developed a liquid retinal prosthesis with less invasive implantation, wide retinal coverage, and high spatial resolution. In this study, the nanoparticles of conjugated semiconducting polymer (poly [3-hexylthiophene], P3HT NPs) were subretinally injected in a rat model of retinitis pigmentosa. P3HT NPs diffuse throughout the entire subretinal space, imitating the spatial distribution of PRs and generating a light-sensitive interface with inner retinal neurons upon single nanoparticles administration [634].

Despite all the progress made, patiants could only expect an artificial vision rather than vision restoration [635]. Significant challenges must be addresses to restore sight. Sufficient amount of visual information must be transmitted from the retina to the brain and interepratation of the artificial visual information requires more advanced image processing techniques [636]. Electrode sizes must be reduced and biocompatibility of the embedded materials with the neural tissue must be enhanced [637]. Though, research in artificial vision is a pioneering research activity in the medical field. One example is the the chemical retinal prosthesis, which pixelated release of neurotransmitter would use as RGC stimulation and has a long priod of preliminary works. To date, there is no report of a functioning one in animal models. However, it has several potential advantages over electrical prosthesis including, the capacity to slow the processes driving remodelling and possibility of corelease of other chemical agents (e.g., trophic factors) [638]. With the progress in material and microfabrication mechanisms now it seems the time to test the first chemical retinal prosthesis. On the other hand, lateral geniculate nucleus (LGN) microstimulation in animal models generated simple discrete visual percepts in a very few preclinical studies which makes it a feasible candidate for future [639, 640].

## Conclusion and future prospects

Photoreceptor degenerations represent a group of multifactorial diseases that result in the common underlying problem of PR loss and irreversible visual deficits. This review has attempted to summarise the most promising treatment strategies in detail, even though at present few interventions have been approved cure for use in these disorders. Recent research findings focused on the early stage of visual disorders have shown that solutions offered by PR neuroprotection hold much promise for future clinical advances. However, early detection of degeneration is important for the optimal implementation of neuroprotective modalities in clinical practice. As such, enhanced screening methods such as autofluorescence of flavoproteins, which permits detection of cellular metabolic dysfunction in the retina, will likely play an important role [641, 642]. Similarly, an understanding of underlying molecular mechanisms will give rise to novel neuroprotective strategies. Recently, metabolic reprogramming using enzymes involved in glycolysis or [251, 643] genetically reprogramming glycolysis [644] have improved overall PR survival. Likewise, some signal transductions may fundamentally regulate PR neuroprotection. Undoubtedly, the future of PR neuroprotection relies on elucidating the mechanisms of the signalling networks involved.

Accordingly, investigation of neuroprotective compounds, metabolic reprogramming, and signal transduction may provide solutions needed to overcome current limitations and lead to highly efficient PR neuroprotective protocols in the near future. Gene therapy is another promising strategy that has potential in the early stages of PR loss. AAV mediated gene delivery has been shown to offer the possibility of stable gene expression as a result of its low immunogenicity and have been used as vectors in several gene therapy trials for retinal degenerations. Also, non-viral vectors and methods, such as electrotransfection, have been employed for transferring gene constructs [645]. In addition to vectors, genome editing by the CRISPER/Cas9 technique shows promise for ocular therapy because of its ability to change or replace genes correctly [95]. While it is expected that early-stage of PR degeneration might be treated with gene therapy, treatment of severely affected retinas in an advanced stage of the disease would likely require a combination of gene therapy together with additional strategies. Presented reports from the Paris Vision Institute and Germany clarified that cell and gene approaches would be synergistic to each other since functional outer segments are rarely found in transplanted PRs. In the late stage of retinal disorders, the transfection of PRs with microbial opsin followed by the introduction of the corrected cells into the subretinal space would provide a novel approach to visual restoration [646]. As mentioned before, a large number of transplanted cells do not pass through the outer limiting membrane and are stopped as the cell clump in the subretinal space [647]. These cells also receive some undesirable signals derived from an unhealthy microenvironment which might affect the final transplanted cell fate [648, 649]. Therefore, in PR degeneration, in which a variety of pathological aspects are involved. As such, vascular alterations culminating is another phenomena affecting the current therapeutic approaches by interruping cell signalling to the brain. Therefore, a combination of a wide range of therapeutic approaches is suggested [507]. Few investigations have been undertaken into the simultaneous injection of multiple cell types to provide both cell source replacement and neurotropic agents that might improve the overall efficacy of transplantation. One example would be hRPCs differentiation and functionality, as measured by ERG, being improved using combined cell transplantation with hBMSCs, which also resulted in suppression of gliosis and microglial activation [650]. Intravitreal administration of combined hematopoietic and mesodermal cells derived from iPSCs could improve vessel formation in diabetic retinopathy based on anti-inflammatory and antioxidative effects (NCT03403699). Different administrative routes for MSC use might also advance the therapeutic outcomes. For instance, intravitreal injection of MSC in combination with subretinal administration of MSCs reduced the immunoreactivity and could be more effective as adjuvant therapy for future studies [651]. Therefore, it seems that the combination of different effective factors provided by various cell sources might improve the efficiency of cell therapy in the future. Moreover, utilizing visual prosthesis, which allows low vision patients to identify infrared radiation and photothermal stimulations emitting from potential hazards such as hot drinks or an open fire, could determine future directions of this topic.

Although much work remains to overcome current limitations impacting the most significant therapeutic strategies, including neuroprotection, gene and cell therapies, continuing research in the field should pave the way to the future restoration of vision in patients with progressive PR degeneration. It seems likely that a combination of neuroprotective pharmacologic solutions, carefully-targeted gene therapies, and clinically safe cell transplantation approaches could generate synergistic clinical outcomes well beyond what is currently available to patients.
